# Supplementary material for: Isolation and Characterization of Galloylglucoses Effective against Multidrug-Resistant Strains of Escherichia coli and Klebsiella pneumoniae
Source: Molecules. 2022 Aug 8;27(15):5045. doi: 10.3390/molecules27155045 (PMC9370434; doi:10.3390/molecules27155045)
Supplement: Supplementary file 1 [file molecules-27-05045-s001.zip › molecules-1839335-supplementary.pdf]

Supporting information for:

Isolation and Characterization of Galloylglucoses Effective against Multidrug-Resistant Strains of *Escherichia coli* and *Klebsiella pneumoniae*.

Nelson E. Masota[1,2], Knut Ohlsen[3], Curd Schollmayer[1], Lorenz Meinel[1] and Ulrike Holzgrabe*[1]

[1]Institute of Pharmacy and Food Chemistry, University of Wuerzburg, Wuerzburg Germany.

[2]School of Pharmacy, Muhimbili University of Health and Allied Sciences, Dar es Salaam, Tanzania.

[3]Institute for Molecular Infection Biology, University of Wuerzburg, Wuerzburg Germany

List of Tables and Figures

Table S1: Summary of preparative HPLC parameters for isolation and purification of subfractions/compounds from the purified water fraction. 4

Figure S1: UV chromatogram subfraction D 5

Figure S2: UV chromatogram of subfraction E 5

Figure S3: Infrared spectrum of 1,2,3,4,6-penta-O-galloyl-β-D-glucose (NMA2) 6

Figure S4: Infrared spectrum of 3-O-digalloyl-1,2,4,6-tetra-O-galloyl-β-D-glucose (NMB4) 6

Figure S4: Infrared spectrum of 6-O-digalloyl-1,2,3,4-tetra-O-galloyl-β-D-glucose (NMB6) 7

Figure S6: Infrared spectrum of 3,6-bis-O-digalloyl-1,2,4-tri-O-galloyl-β-D-glucose (NMC3) 7

Figure S8: HR-ESI-MS spectra of 3-O-digalloyl-1,2,4,6-tetra-O-galloyl-β-D-glucose (NMB4) showing the full mass spectrum (A), zoom in of the major peak region (B) and simulated spectrum for the shown molecular formula (C). 8

Figure S9: HR-ESI-MS spectra of 6-O-digalloyl-1,2,3,4-tetra-O-galloyl-β-D-glucose (NMB6) showing the full mass spectrum (A), zoom in of the major peak region (B) and simulated spectrum for the shown molecular formula (C). 9

Figure S10: HR-ESI-MS spectra of 3,6-bis-O-digalloyl-1,2,4-tri-O-galloyl-β-D-glucose (NMC3) showing the full mass spectrum (A), zoom in of the major peak region (B) and simulated spectrum for the shown molecular formula (C). 9

Figure S11: ^{1}H spectrum of 1,2,3,4,6-penta-O-galloyl-β-D-glucose (NMA2) 10

Figure S12: ^{13}C spectrum of 1,2,3,4,6-penta-O-galloyl-β-D-glucose (NMA2) 10

Figure S13: DEPT- 135 spectrum of 1,2,3,4,6-penta-O-galloyl-β-D-glucose (NMA2) 11

Figure S14: COSY spectrum of 1,2,3,4,6-penta-O-galloyl-β-D-glucose (NMA2) 11

Figure S15: HSQC spectrum of 1,2,3,4,6-penta-O-galloyl-β-D-glucose (NMA2)...... 12
Figure S16: Zoom in of the aromatic aromatic region of HSQC spectrum of 1,2,3,4,6-penta-O-galloyl-β-D-glucose (NMA2)... 12
Figure S17: HMBC spectrum of 1,2,3,4,6-penta-O-galloyl-β-D-glucose (NMA2)...... 13
Figure S18: Zoom in of the aromatic region of HMBC spectrum of 1,2,3,4,6-penta-O-galloyl-β-D-glucose (NMA2)... 13
Figure S19: 1H spectrum of 3-O-digalloyl-1,2,4,6-tetra-O-galloyl-β-D-glucose (NMB4)... 14
Figure S20: 13C spectrum of 3-O-digalloyl-1,2,4,6-tetra-O-galloyl-β-D-glucose (NMB4)... 14
Figure S21: DEPT-135 of 3-O-digalloyl-1,2,4,6-tetra-O-galloyl-β-D-glucose (NMB4) 15
Figure S22: COSY Spectrum of 3-O-digalloyl-1,2,4,6-tetra-O-galloyl-β-D-glucose (NMB4)... 15
Figure S23: HSQC spectrum of 3-O-digalloyl-1,2,4,6-tetra-O-galloyl-β-D-glucose (NMB4)... 16
Figure S24: Zoom in of the aromatic region of HSQC spectrum of 3-O-digalloyl-1,2,4,6-tetra-O-galloyl-β-D-glucose (NMB4)... 16
Figure S25: HMBC spectrum of 3-O-digalloyl-1,2,4,6-tetra-O-galloyl-β-D-glucose (NMB4)... 17
Figure S26: Zoom in of the aromatic region of HMBC spectrum of 3-O-digalloyl-1,2,4,6-tetra-O-galloyl-β-D-glucose (NMB4)... 17
Figure S27: 1H spectrum of 6-O-digalloyl-1,2,3,4-tetra-O-galloyl-β-D-glucose (NMB6)... 18
Figure S28: 13C spectrum of 6-O-digalloyl-1,2,3,4-tetra-O-galloyl-β-D-glucose (NMB6)... 18
Figure S29: DEPT135 spectrum of 6-O-digalloyl-1,2,3,4-tetra-O-galloyl-β-D-glucose (NMB6)... 19
Figure S30: COSY spectrum of 6-O-digalloyl-1,2,3,4-tetra-O-galloyl-β-D-glucose (NMB6)... 19
Figure S31: HSQC spectrum of 6-O-digalloyl-1,2,3,4-tetra-O-galloyl-β-D-glucose (NMB6)... 20
Figure S32: Zoom in of the aromatic region of HSQC spectrum of 6-O-digalloyl-1,2,3,4-tetra-O-galloyl-β-D-glucose (NMB6)... 20
Figure S33: HMBC spectrum of 6-O-digalloyl-1,2,3,4-tetra-O-galloyl-β-D-glucose (NMB6)... 21
Fig S34: Zoom in of the aromatic region of HMBC spectrum of 6-O-digalloyl-1,2,3,4-tetra-O-galloyl-β-D-glucose (NMB6) 21
Figure S35: ^{1}H spectrum of 3,6-bis-O-digalloyl-1,2,4-tri-O-galloyl-β-D-glucose (NMC3)... 22

Figure S36: ^{13}C spectrum of 3,6-bis-O-digalloyl-1,2,4-tri-O-galloyl-β-D-glucose (NMC3) 22
Figure S37: DEPT-135 spectrum of 3,6-bis-O-digalloyl-1,2,4-tri-O-galloyl-β-D-glucose (NMC3) 23
Figure S38: COSY spectrum of 3,6-bis-O-digalloyl-1,2,4-tri-O-galloyl-β-D-glucose (NMC3) 23
Figure S39: HSQC spectrum of 3,6-bis-O-digalloyl-1,2,4-tri-O-galloyl-β-D-glucose (NMC3) 24
Figure S40: Zoom in of the aromatic region of the HSQC spectrum of 6-O-digalloyl-1,2,3,4-tetra-O-galloyl-β-D-glucose (NMB6) 24
Figure S41: HMBC spectrum of 3,6-bis-O-digalloyl-1,2,4-tri-O-galloyl-β-D-glucose (NMC3) 25
Figure S42: Zoom in of the aromatic region of HMBC spectrum of 3,6-bis-O-digalloyl-1,2,4-tri-O-galloyl-β-D-glucose (NMC3) 25

Table S1: Summary of preparative HPLC parameters for isolation and purification of subfractions/compounds from the purified water fraction.

**Fraction to be Refined**	**Stationary phase**	**Mobile phase composition as %v/v of acetonitrile in water and other chromatographic conditions. (UV detection wavelength = 254 nm)**	**Recovered subfractions/ compounds**
Purified water fraction	ZORBAX XDB-C18, 21.2 x 150 mm, 5 µm (Agilent, US)	20 – 20% (0 – 3 min), 20 – 45% (3 – 32 min), 45 – 45% (32 – 33 min), 45 – 95% (33 – 35 min), 95 – 95% (35 – 36 min), 95 – 20% (36 – 38 min).<br><br>Sample conc. = 250 mg/ mL, injection volume = 50 µL, Flow rate = 4 ml/min.	pooled first minor fractions, and major fractions A, B, C, D, E
A		20 – 20% (0 – 29 min), 20 – 50% (29 – 32 min), 50 – 50% (32 – 34 min), 50 – 20% (34 – 36 min), 20 – 20% (36 – 40 min).<br><br>Sample conc. = 100 mg/ mL, injection volume = 100 µL, Flow rate = 4 ml/min.	A1, A2
B		0 – 20% (0 – 32 min), 20 – 75% (32 – 35 min), 75 – 75% (35 – 38 min), 75 – 20% (38 – 41 min), 20 – 20% (41 – 44 min).<br><br>Sample conc. = 22.5 mg/ mL, injection volume = 8 µL, Flow rate = 6 ml/min.	B1, B2, B3, B4&5, B6
C		5 – 5 % (0 – 5 min), 5 – 26% (5 – 7 min), 26 – 26% (7 – 27 min), 26 – 5% (27 – 29 min), 5 – 5% (29 – 31 min).<br><br>Sample conc. = 100 mg/ mL, injection volume = 20 µL, Flow rate = 4 ml/min.	C1, C2, C3
D		31 – 31% (0 – 32 min)<br><br>Sample conc. = 100 mg/ mL, injection volume = 20 µL, Flow rate = 2 ml/min.	D1, D2, D3&4
E		31 – 31% (0 – 35 min)<br><br>Sample conc. = 100 mg/ mL, injection volume = 35 µL, Flow rate = 2 ml/min.	E1, E2, E3&4, E5
B4&5	ZORBAX Eclipse XDB-18, Semi-Preparative 9.4 x 250 mm, 5 µ (Agilent, US)	0 – 17.5% (0 – 34 min), 17.5 – 75% (34 – 37 min), 75 – 75% (37 – 41 min), 75 – 17.5% (41 – 43 min), 17.5 – 17.5 % (29 – 31 min).<br><br>Sample conc. = 13.5 mg/ mL, injection volume = 50 µL, Flow rate = 4 ml/min.	B4, B5

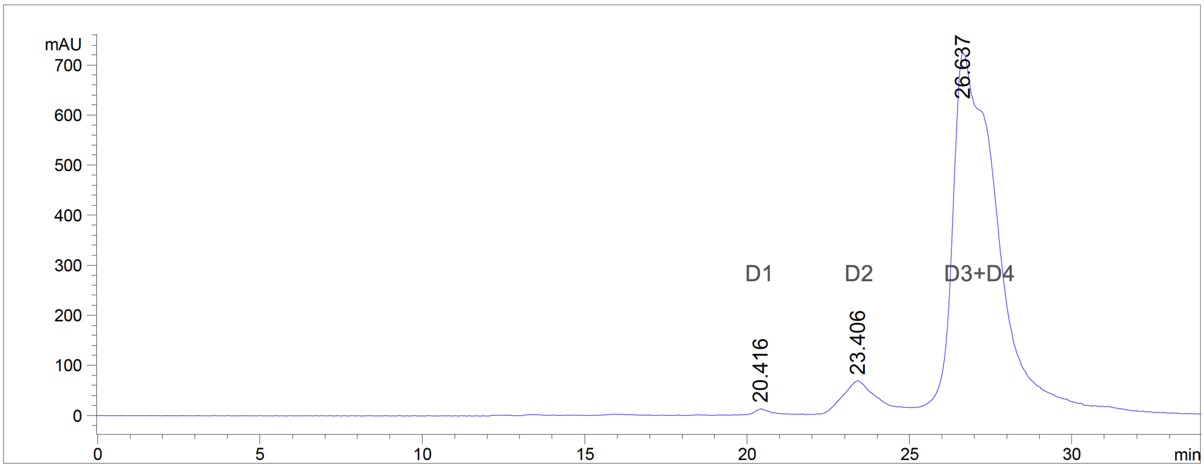

Figure S1: UV chromatogram subfraction D

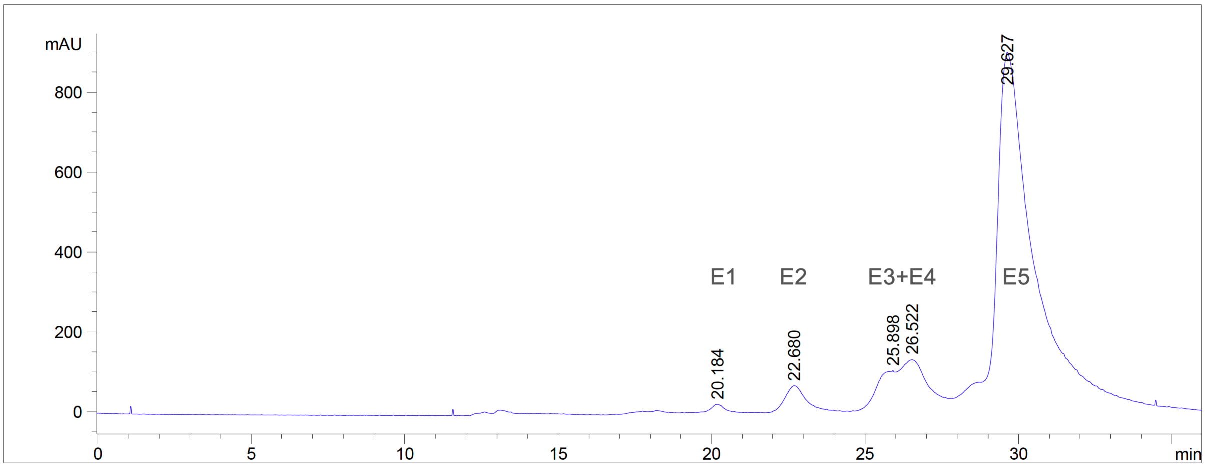

Figure S2: UV chromatogram of subfraction E

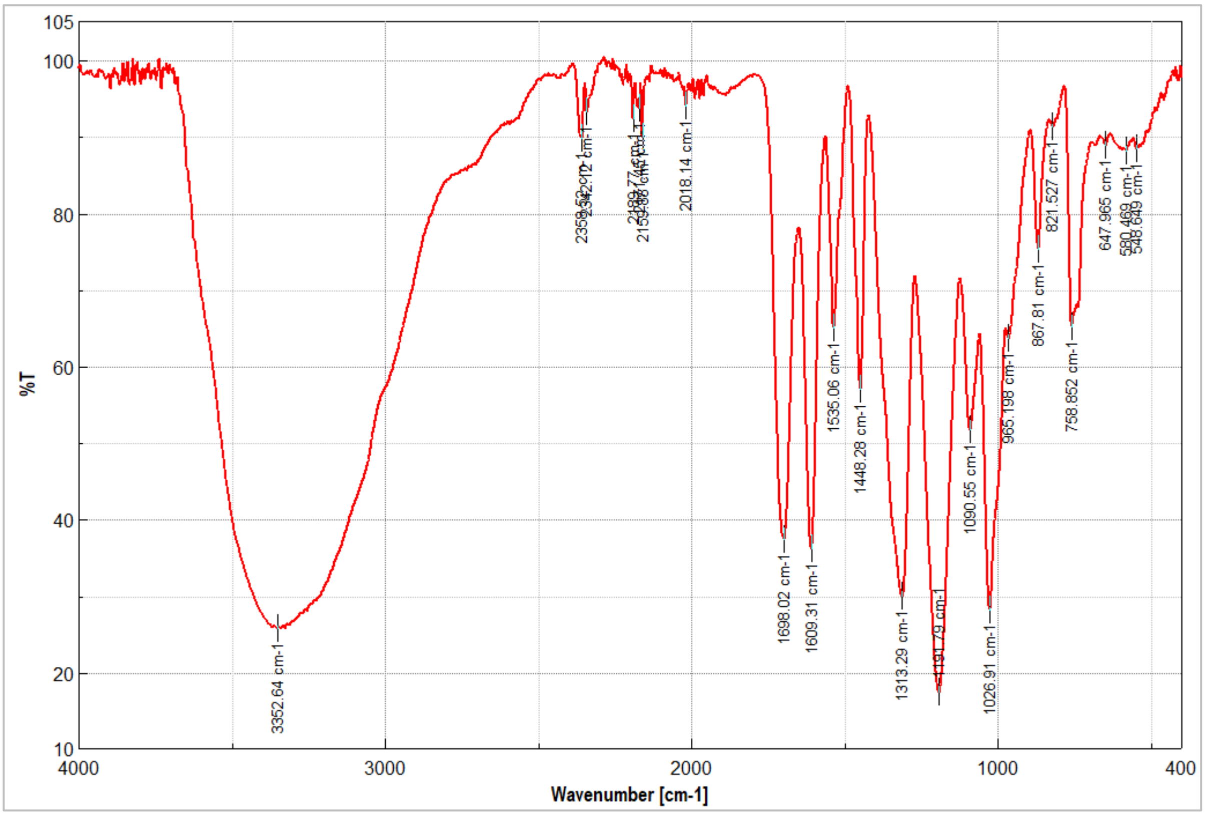

Figure S3: Infrared spectrum of 1,2,3,4,6-penta-O-galloyl-β-D-glucose (NMA2)

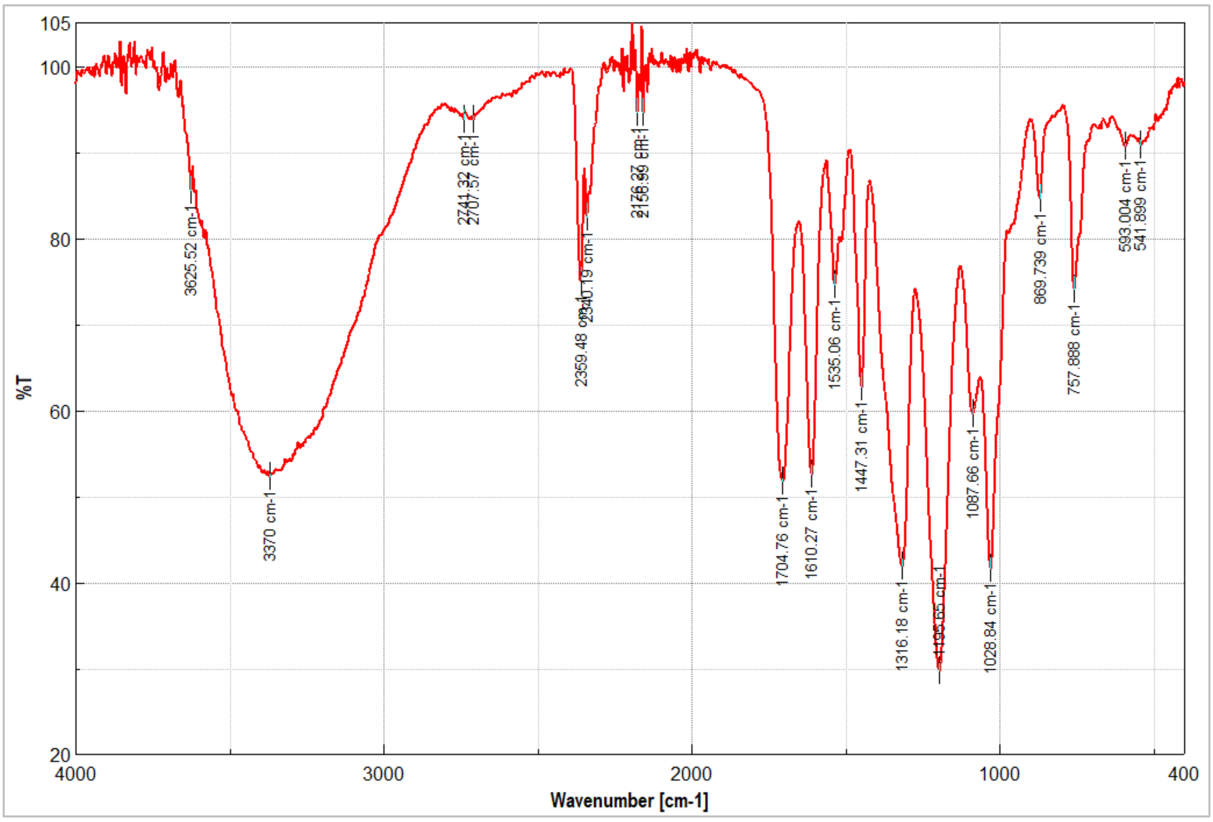

Figure S4: Infrared spectrum of 3-O-digalloyl-1,2,4,6-tetra-O-galloyl-β-D-glucose (NMB4)

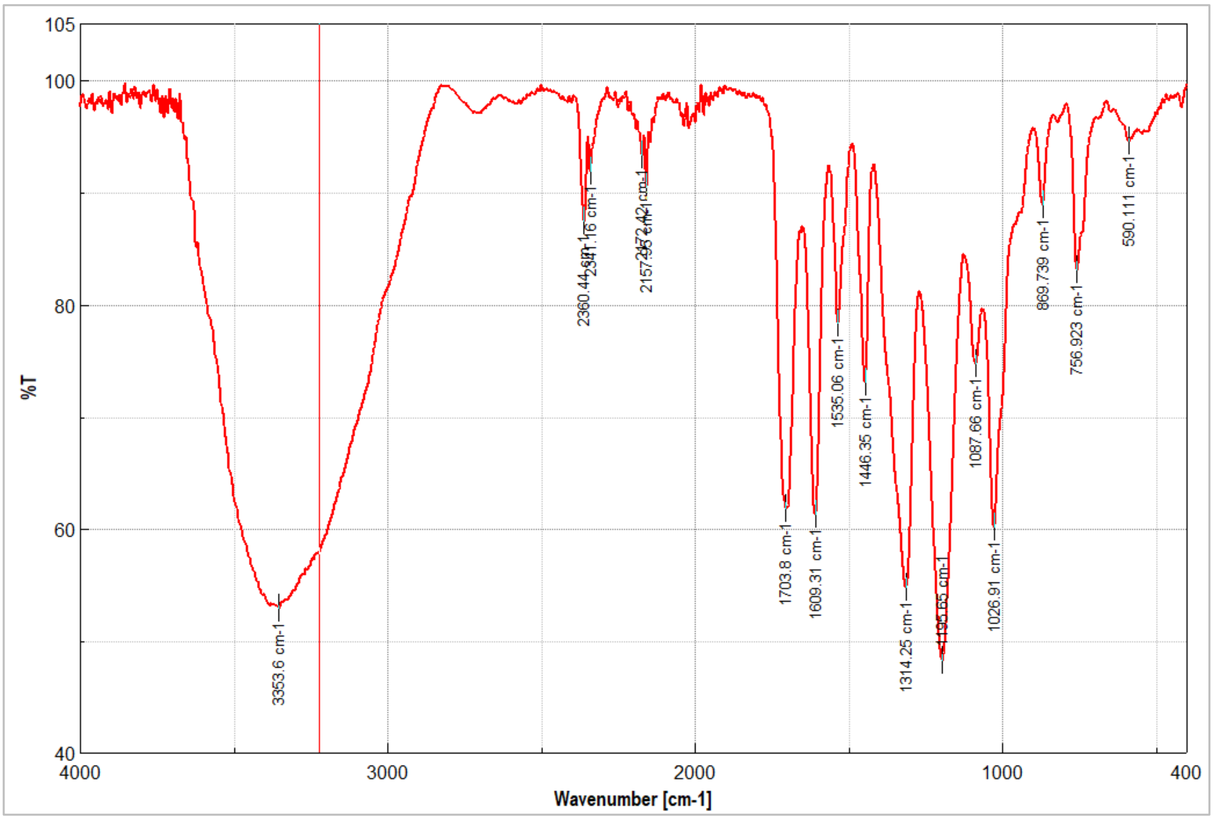

Figure S4: Infrared spectrum of 6-O-digalloyl-1,2,3,4-tetra-O-galloyl-β-D-glucose (NMB6)

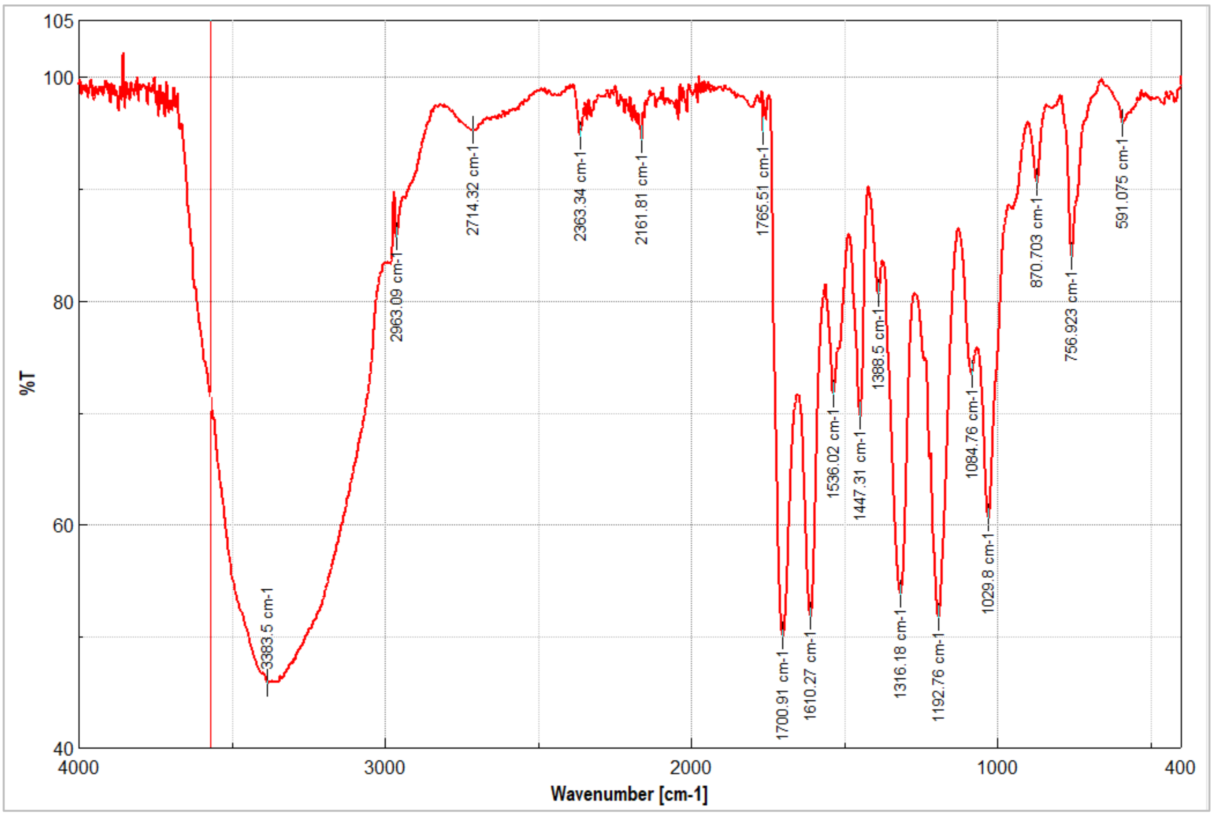

Figure S6: Infrared spectrum of 3,6-bis-O-digalloyl-1,2,4-tri-O-galloyl-β-D-glucose (NMC3)

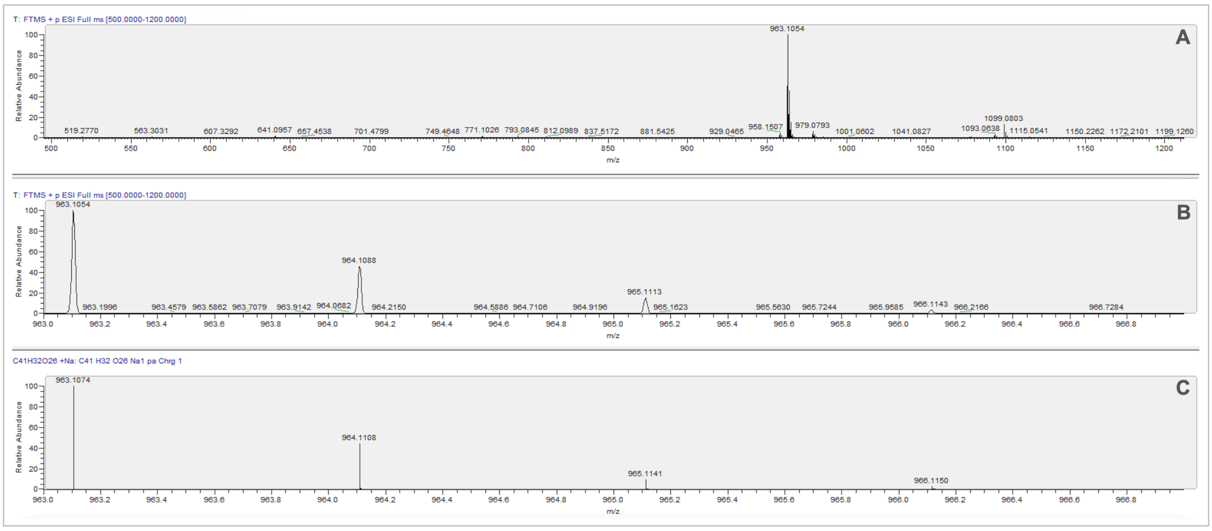

Figure S7: HR-ESI-MS spectra of 1,2,3,4,6-penta-O-galloyl-β-D-glucose (NMA2) showing the full mass spectrum (A), zoom in of the major peak region (B) and simulated spectrum for the shown molecular formula (C).

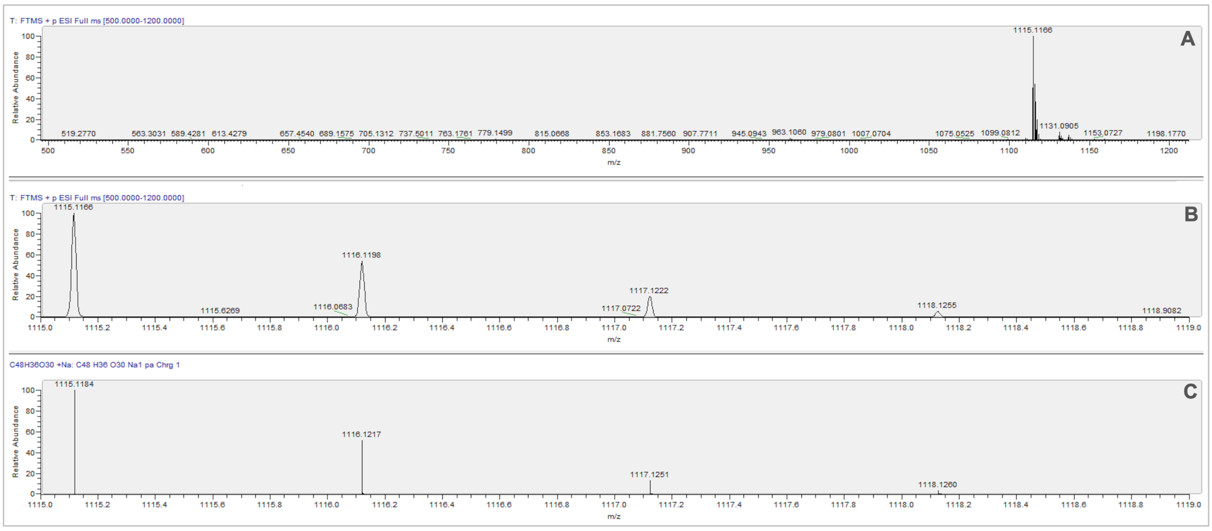

Figure S8: HR-ESI-MS spectra of 3-O-digalloyl-1,2,4,6-tetra-O-galloyl-β-D-glucose (NMB4) showing the full mass spectrum (A), zoom in of the major peak region (B) and simulated spectrum for the shown molecular formula (C).

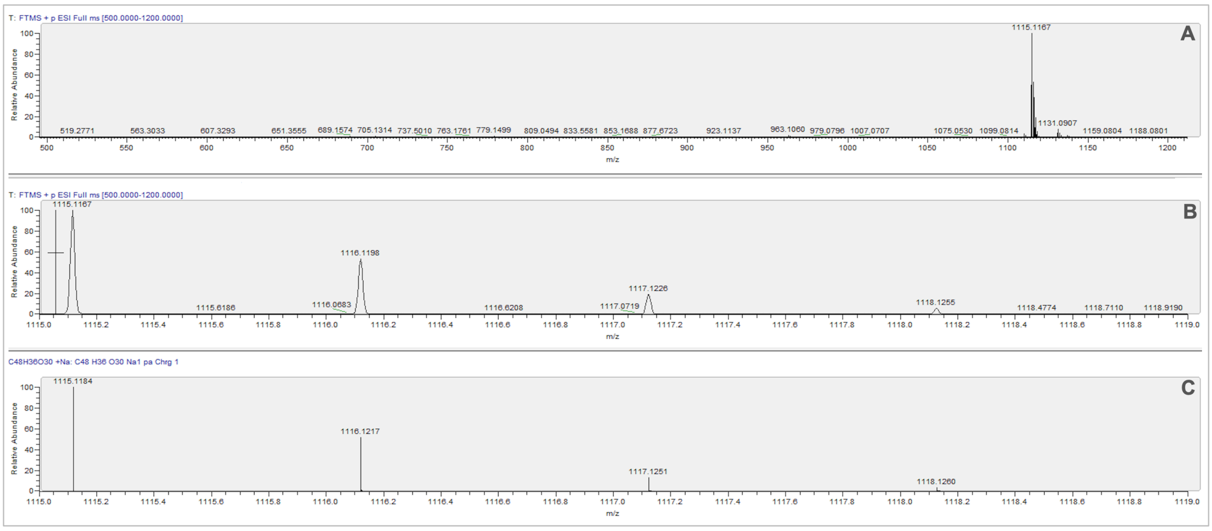

Figure S9: HR-ESI-MS spectra of 6-O-digalloyl-1,2,3,4-tetra-O-galloyl-β-D-glucose (NMB6) showing the full mass spectrum (A), zoom in of the major peak region (B) and simulated spectrum for the shown molecular formula (C).

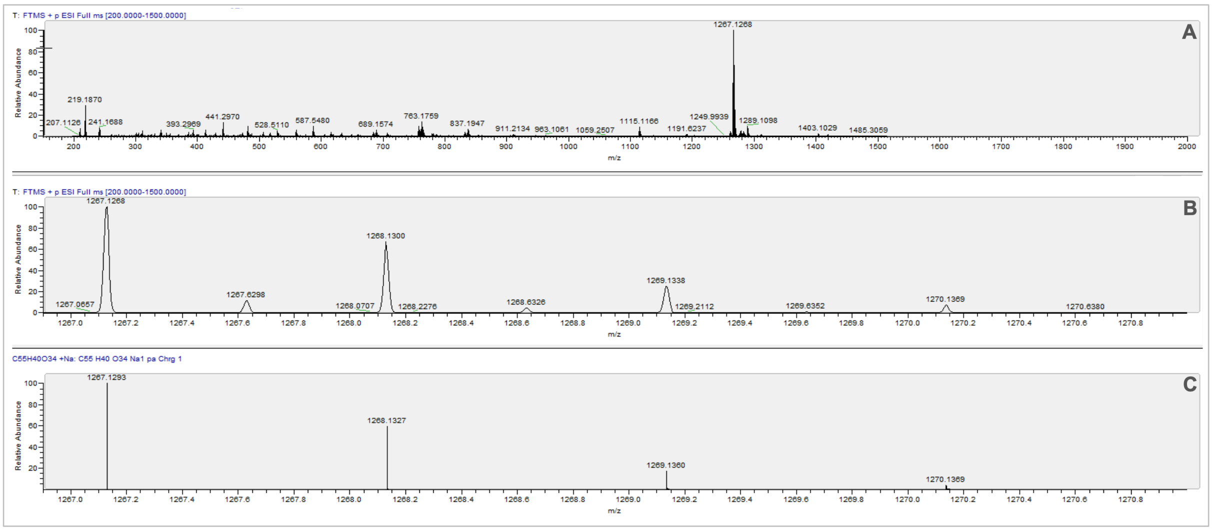

Figure S10: HR-ESI-MS spectra of 3,6-bis-O-digalloyl-1,2,4-tri-O-galloyl-β-D-glucose (NMC3) showing the full mass spectrum (A), zoom in of the major peak region (B) and simulated spectrum for the shown molecular formula (C).

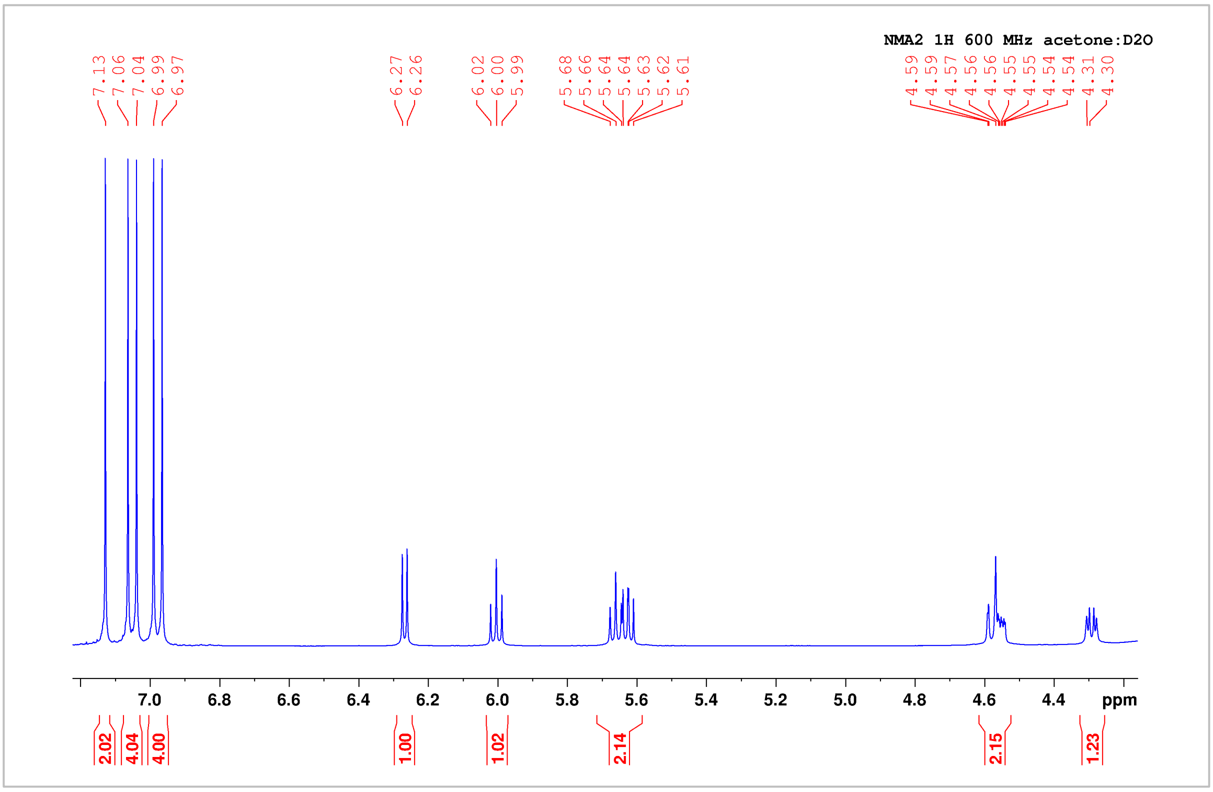

Figure S11: ^{1}H spectrum of 1,2,3,4,6-penta-O-galloyl-β-D-glucose (NMA2)

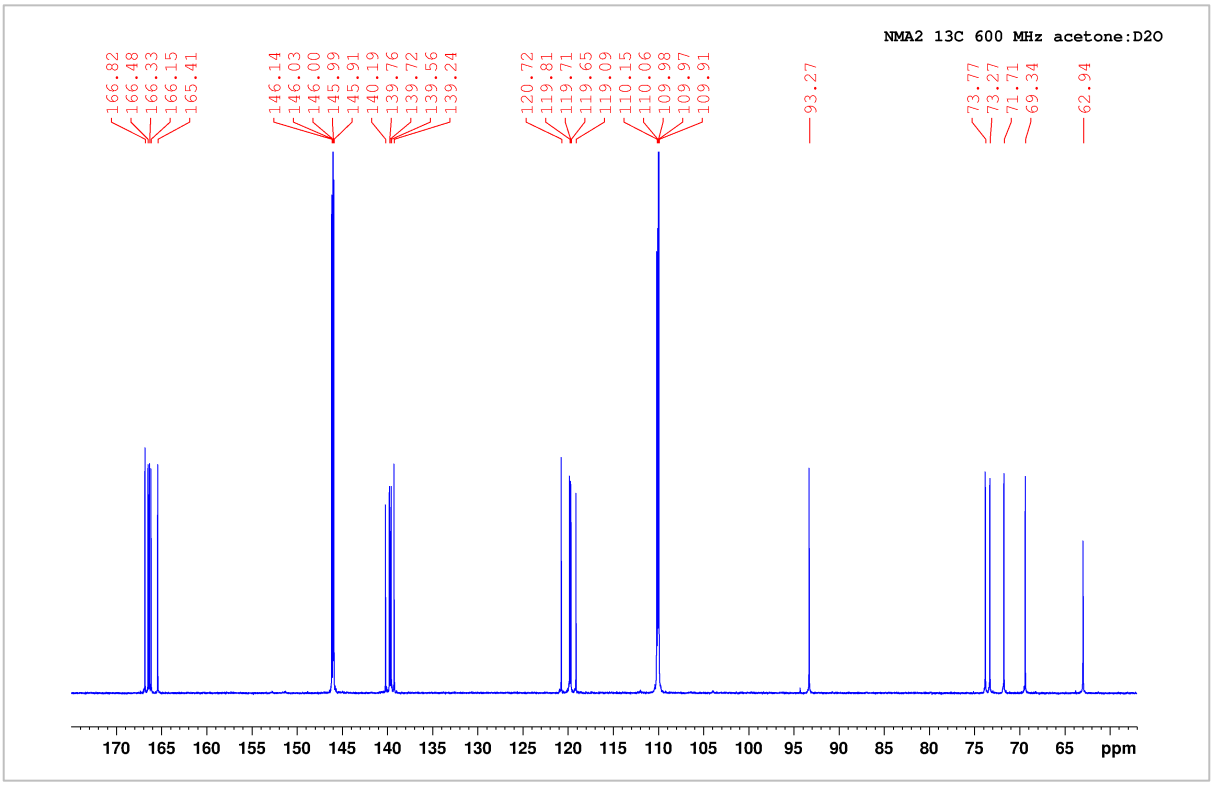

Figure S12: ^{13}C spectrum of 1,2,3,4,6-penta-O-galloyl-β-D-glucose (NMA2)

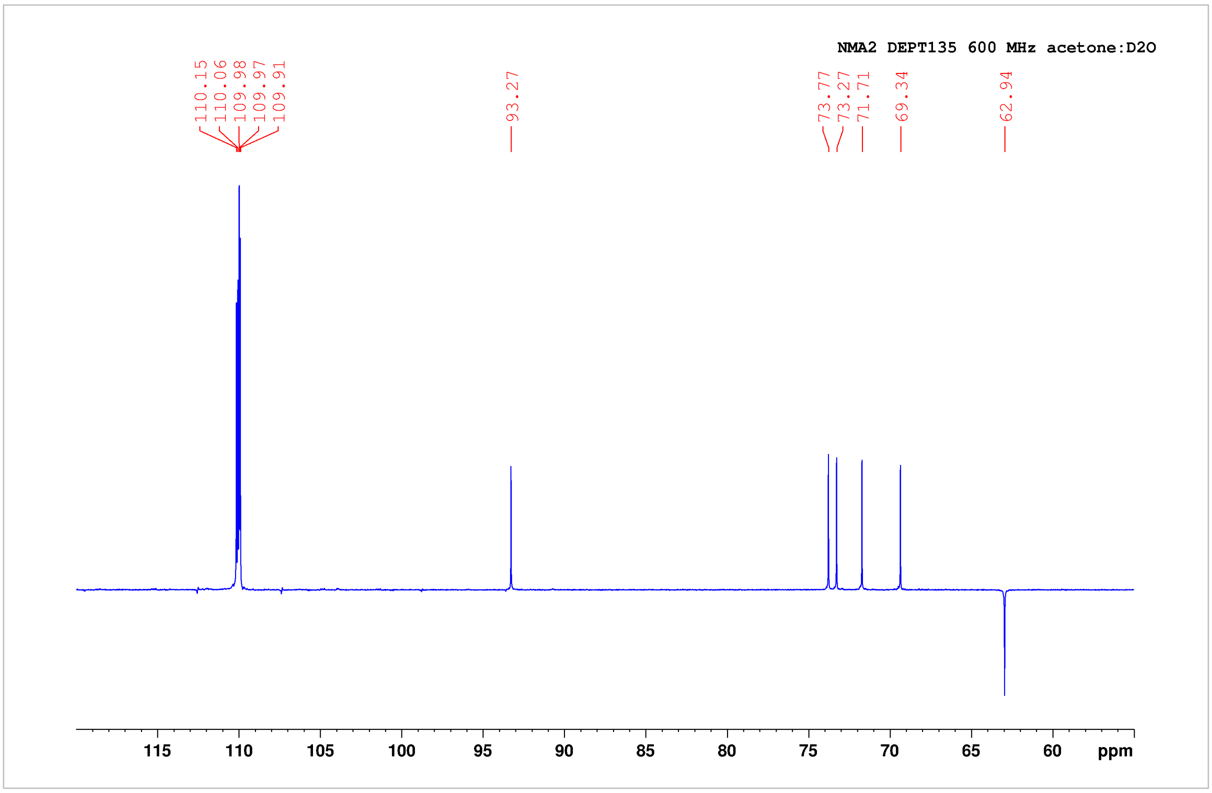

Figure S13: DEPT- 135 spectrum of 1,2,3,4,6-penta-O-galloyl-β-D-glucose (NMA2)

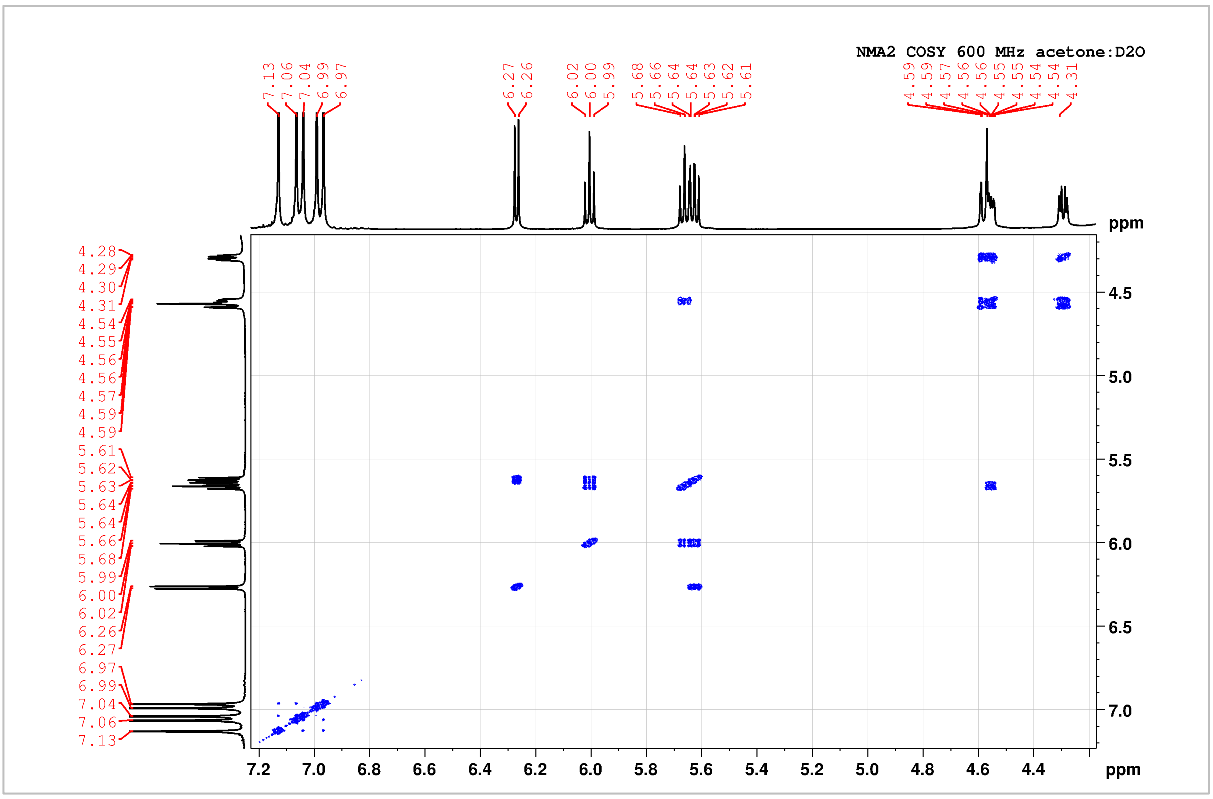

Figure S14: COSY spectrum of 1,2,3,4,6-penta-O-galloyl-β-D-glucose (NMA2)

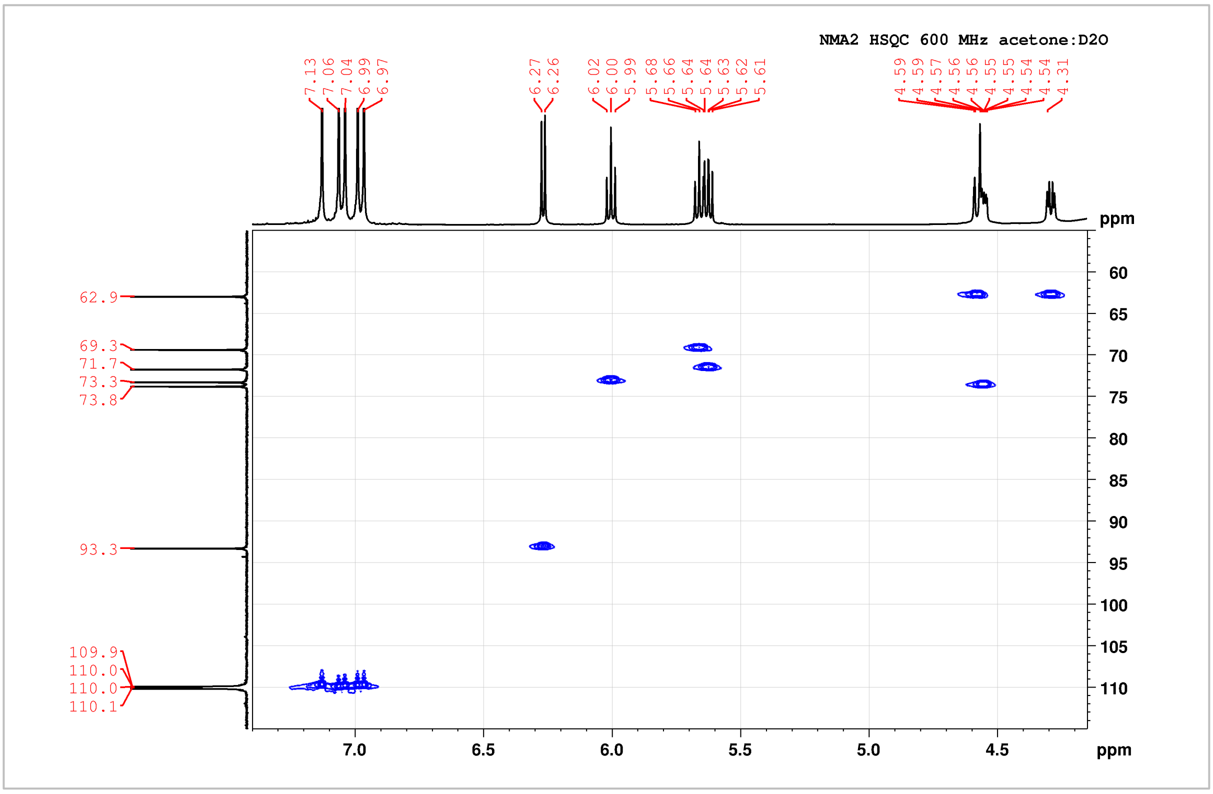

Figure S15: HSQC spectrum of 1,2,3,4,6-penta-O-galloyl-β-D-glucose (NMA2)

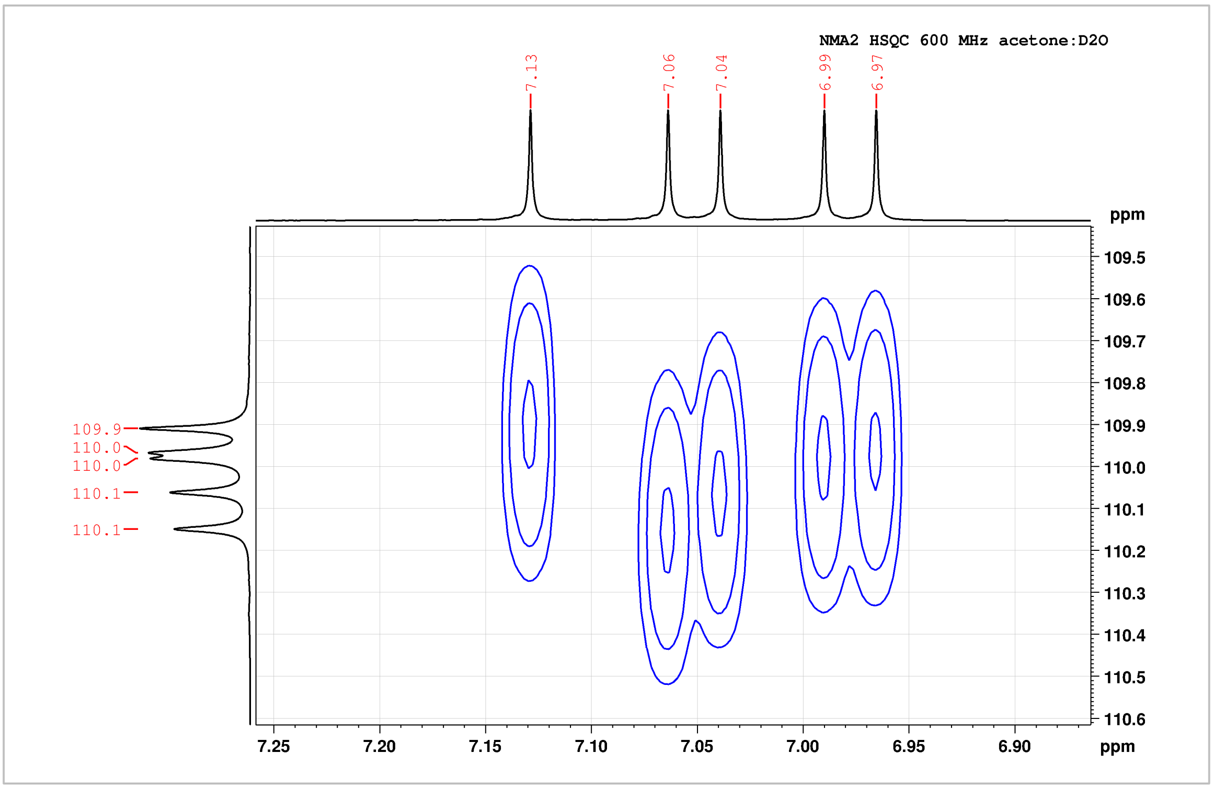

Figure S16: Zoom in of the aromatic aromatic region of HSQC spectrum of 1,2,3,4,6-penta-O-galloyl-β-D-glucose (NMA2)

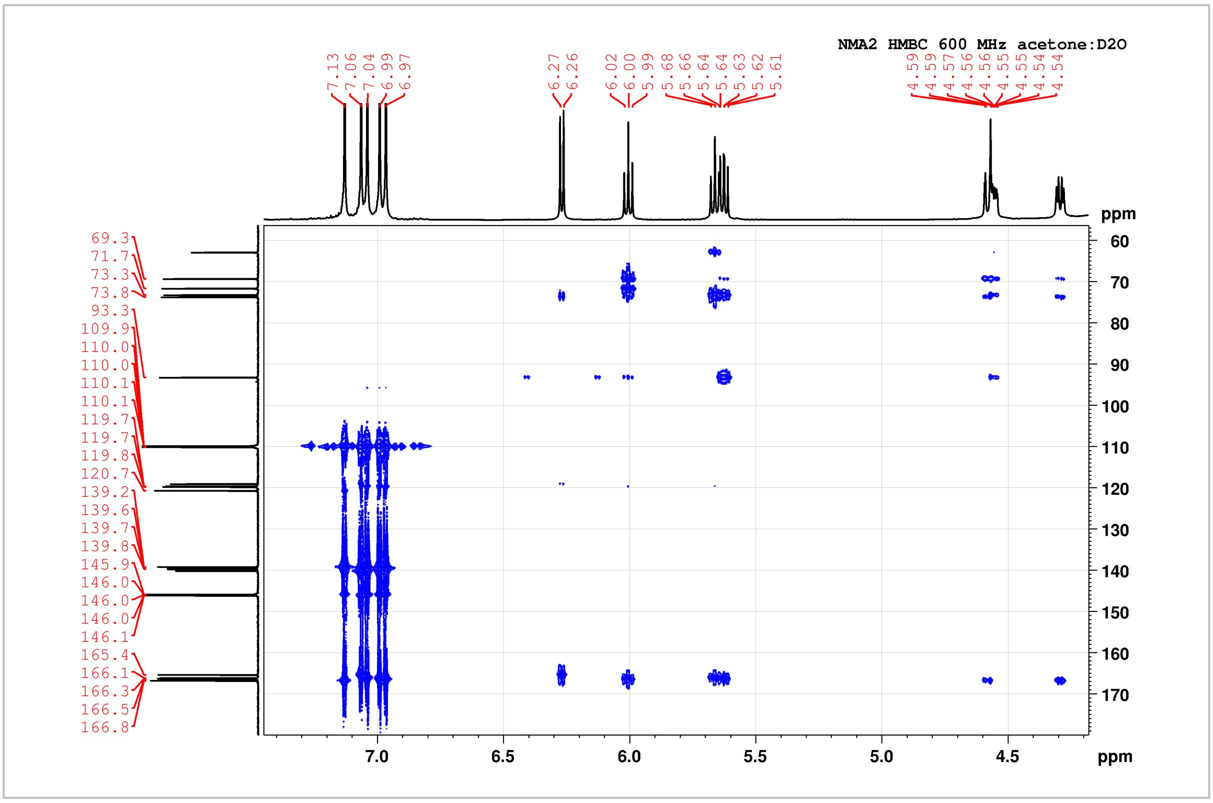

Figure S17: HMBC spectrum of 1,2,3,4,6-penta-O-galloyl-β-D-glucose (NMA2)

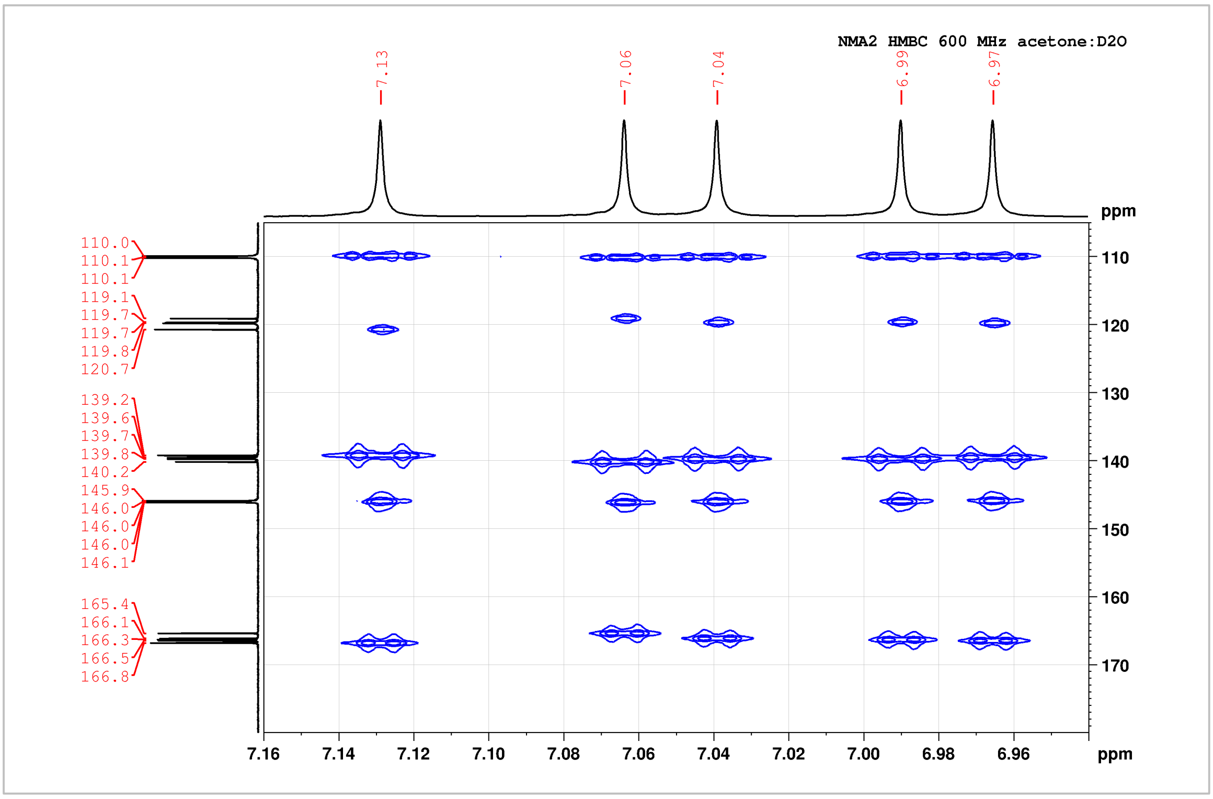

Figure S18: Zoom in of the aromatic region of HMBC spectrum of 1,2,3,4,6-penta-O-galloyl-β-D-glucose (NMA2)

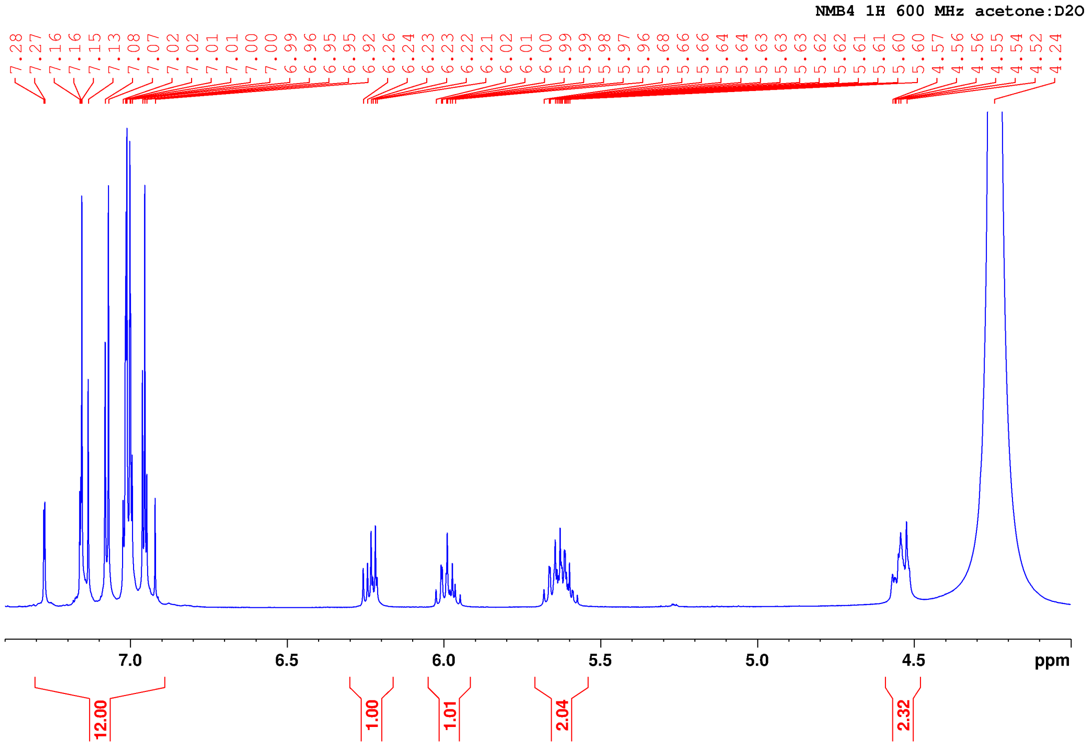

Figure S19: 1H spectrum of 3-O-digalloyl-1,2,4,6-tetra-O-galloyl-β-D-glucose (NMB4)

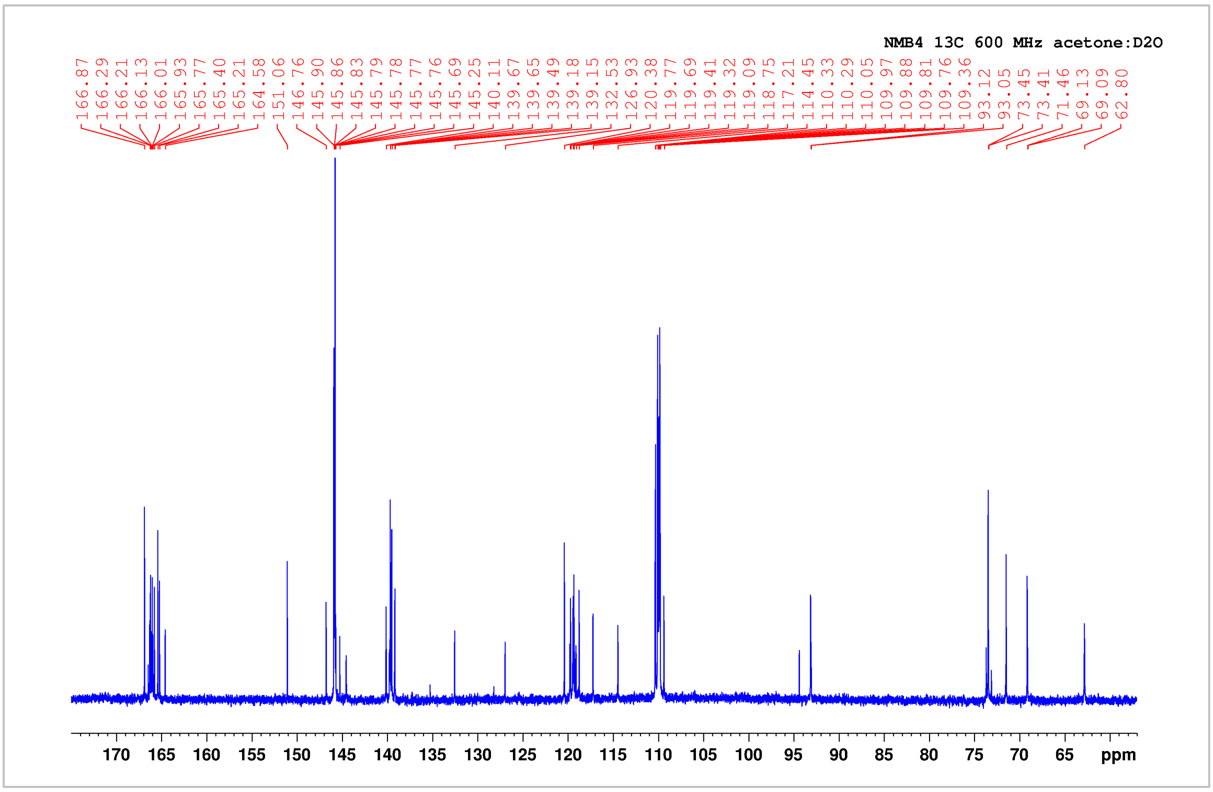

Figure S20: 13C spectrum of 3-O-digalloyl-1,2,4,6-tetra-O-galloyl-β-D-glucose (NMB4)

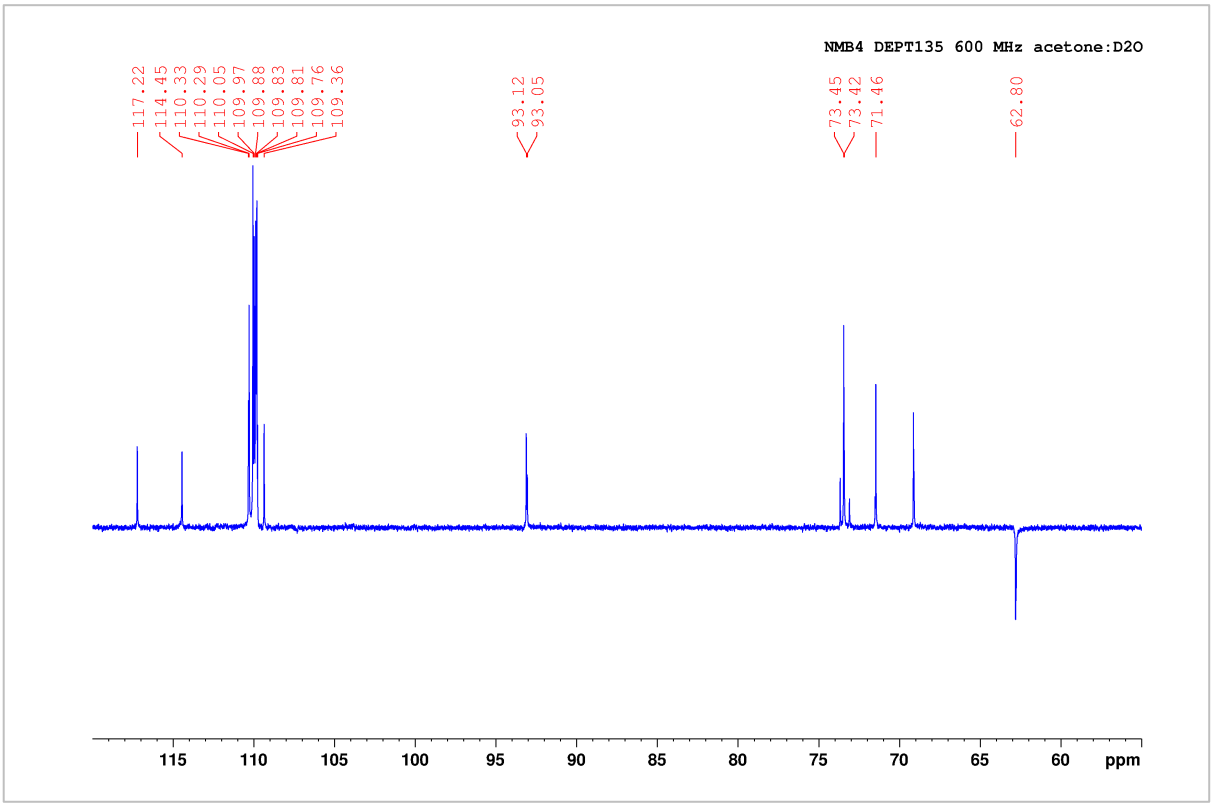

Figure S21: DEPT-135 of 3-O-digalloyl-1,2,4,6-tetra-O-galloyl-β-D-glucose (NMB4)

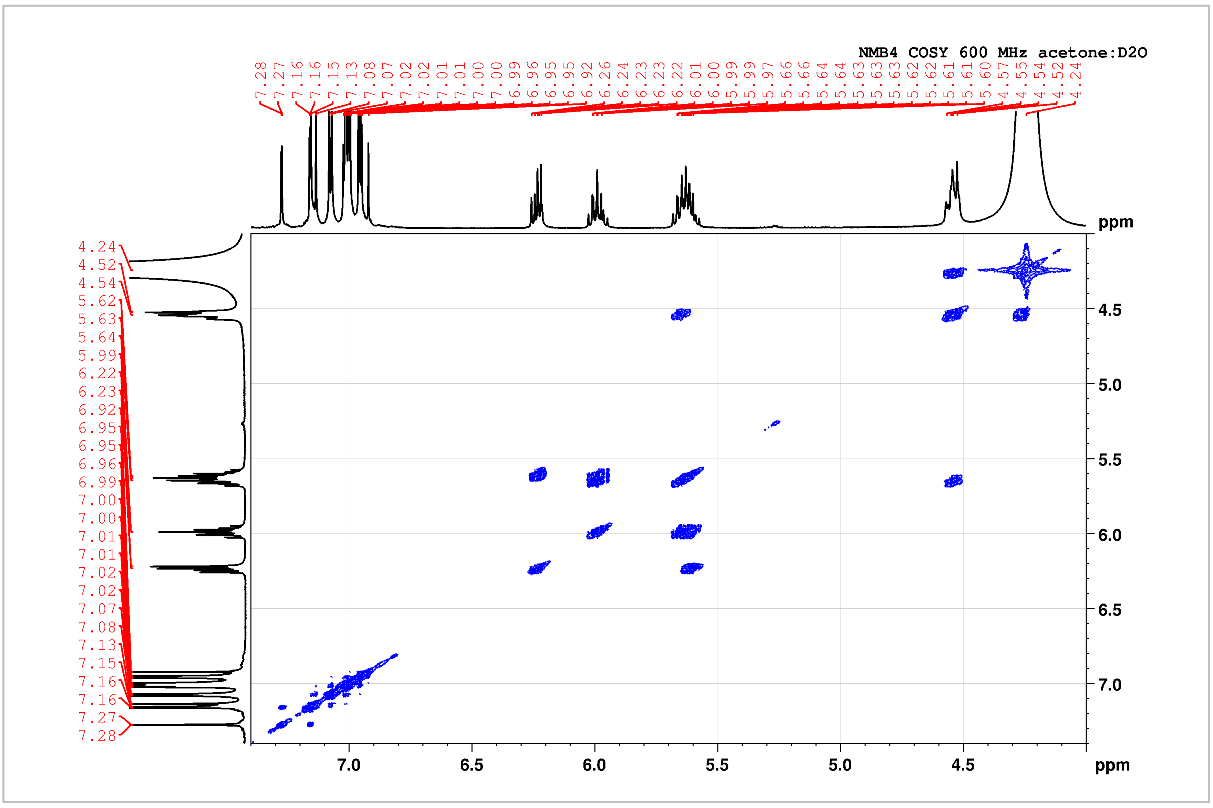

Figure S22: COSY Spectrum of 3-O-digalloyl-1,2,4,6-tetra-O-galloyl-β-D-glucose (NMB4)

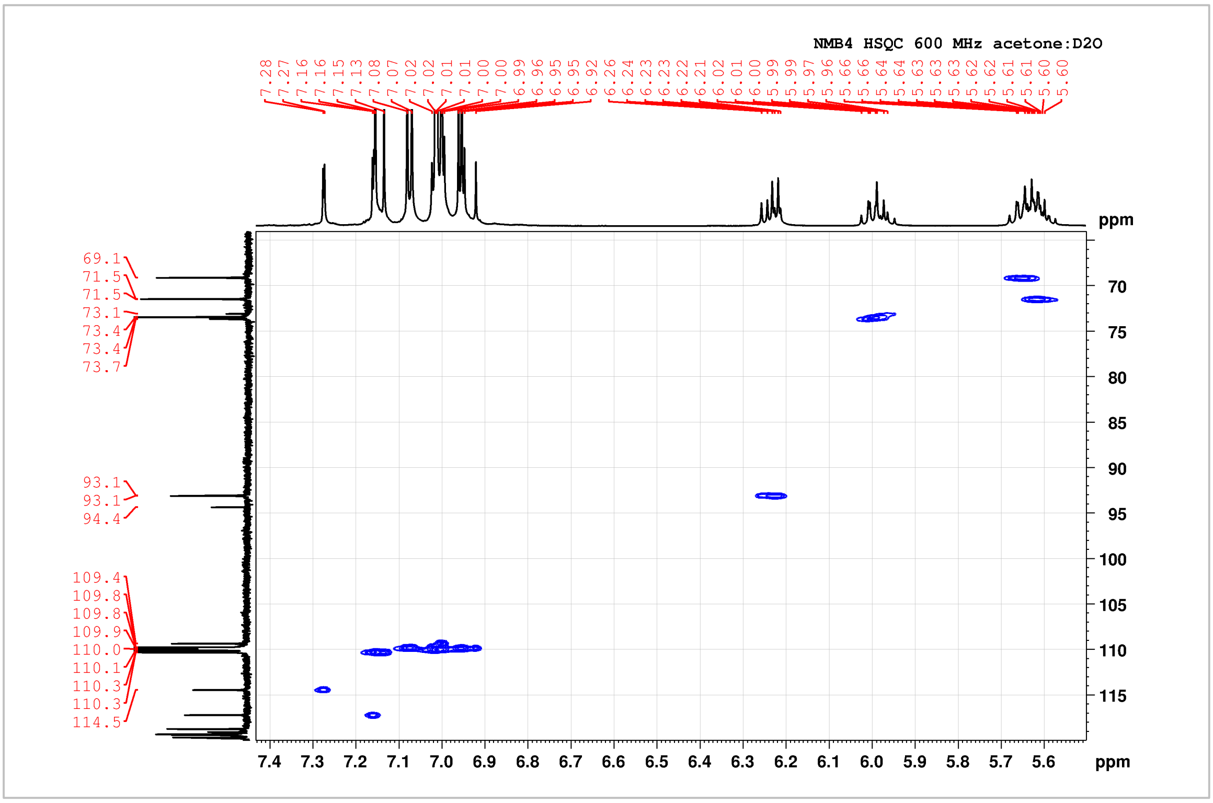

Figure S23: HSQC spectrum of 3-O-digalloyl-1,2,4,6-tetra-O-galloyl-β-D-glucose (NMB4)

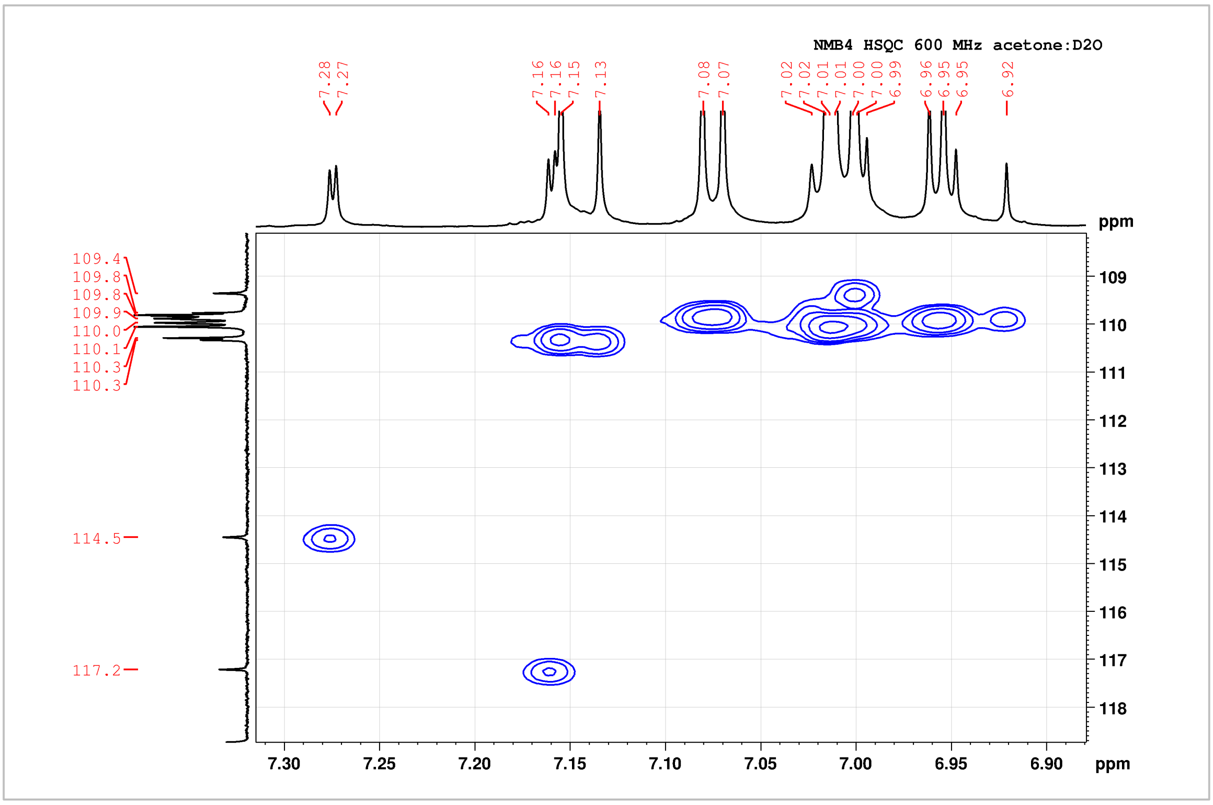

Figure S24: Zoom in of the aromatic region of HSQC spectrum of 3-O-digalloyl-1,2,4,6-tetra-O-galloyl-β-D-glucose (NMB4)

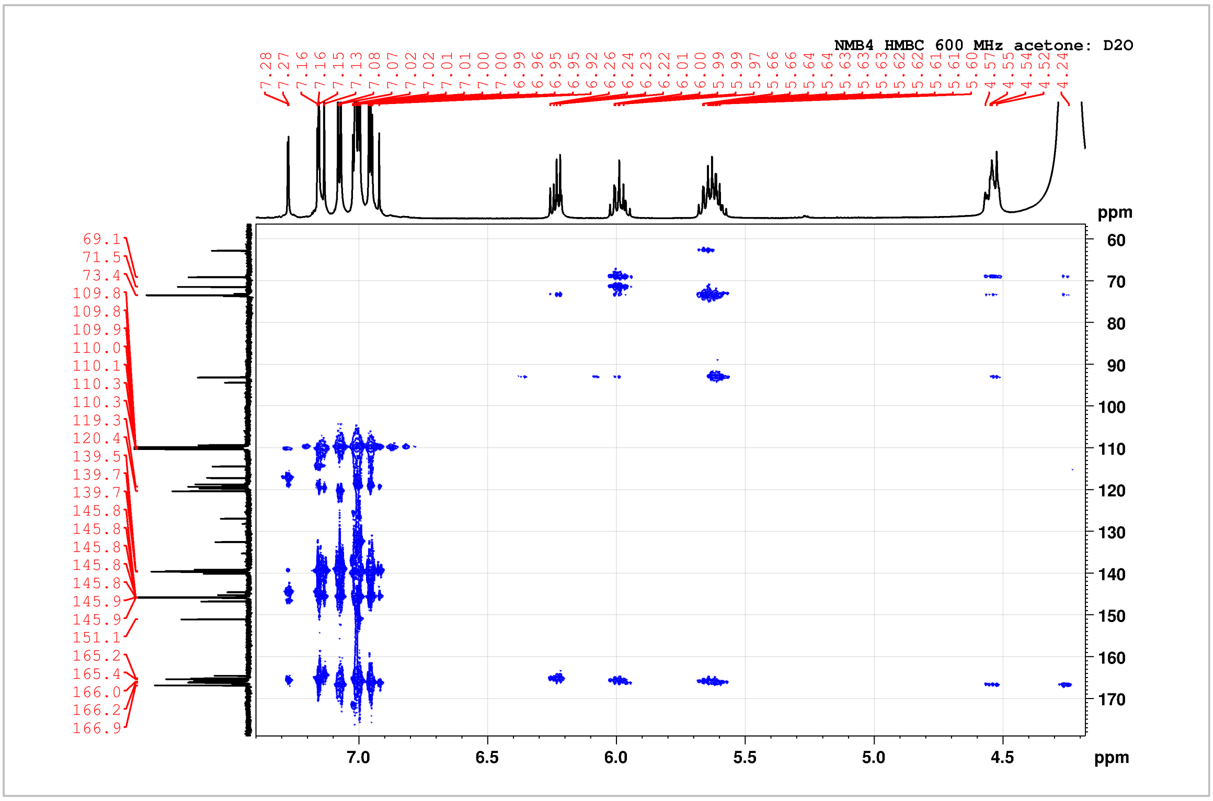

Figure S25: HMBC spectrum of 3-O-digalloyl-1,2,4,6-tetra-O-galloyl-β-D-glucose (NMB4)

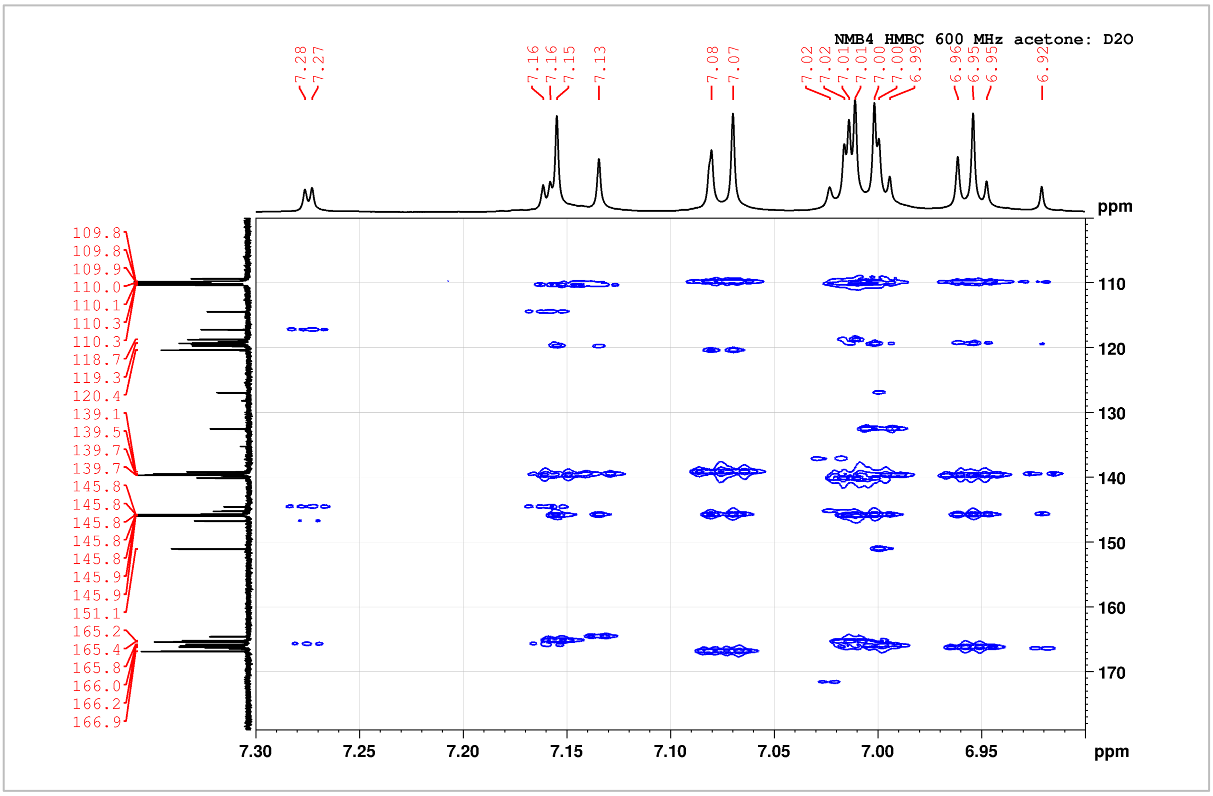

Figure S26: Zoom in of the aromatic region of HMBC spectrum of 3-O-digalloyl-1,2,4,6-tetra-O-galloyl-β-D-glucose (NMB4)

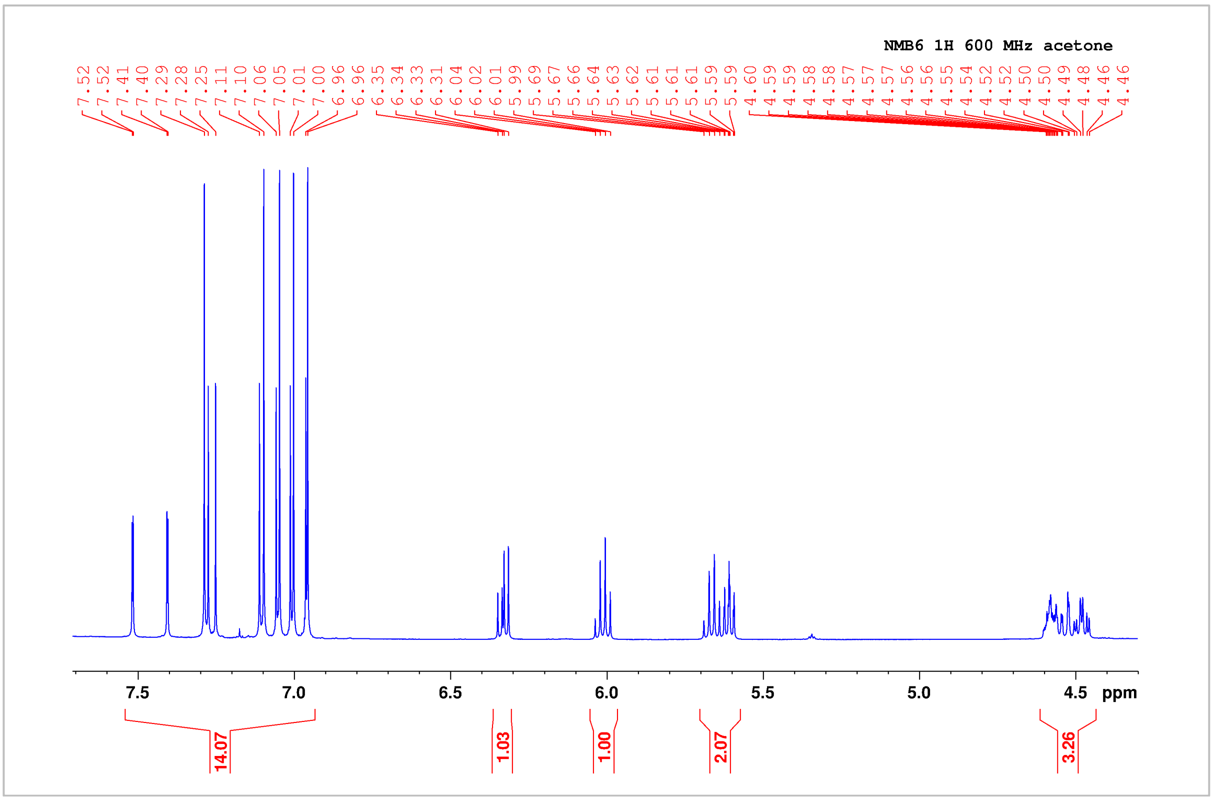

Figure S27: 1H spectrum of 6-O-digalloyl-1,2,3,4-tetra-O-galloyl-β-D-glucose (NMB6)

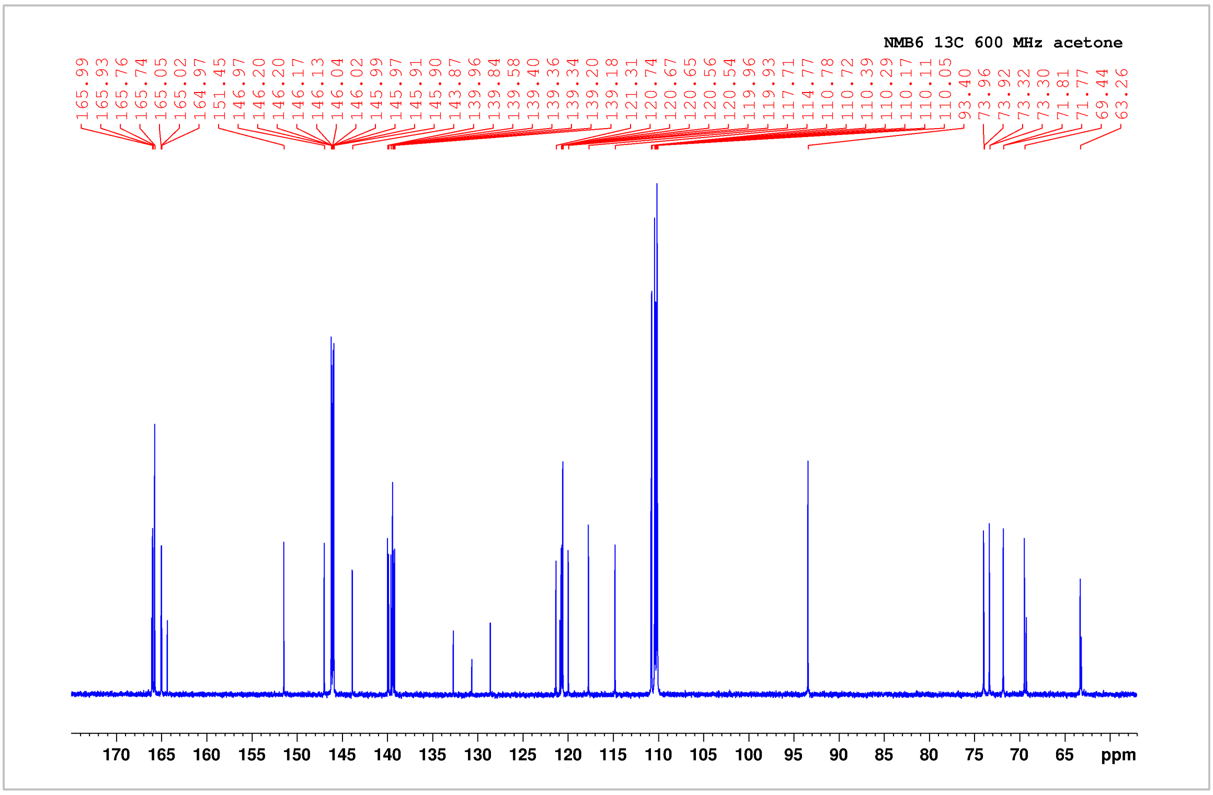

Figure S28: 13C spectrum of 6-O-digalloyl-1,2,3,4-tetra-O-galloyl-β-D-glucose (NMB6)

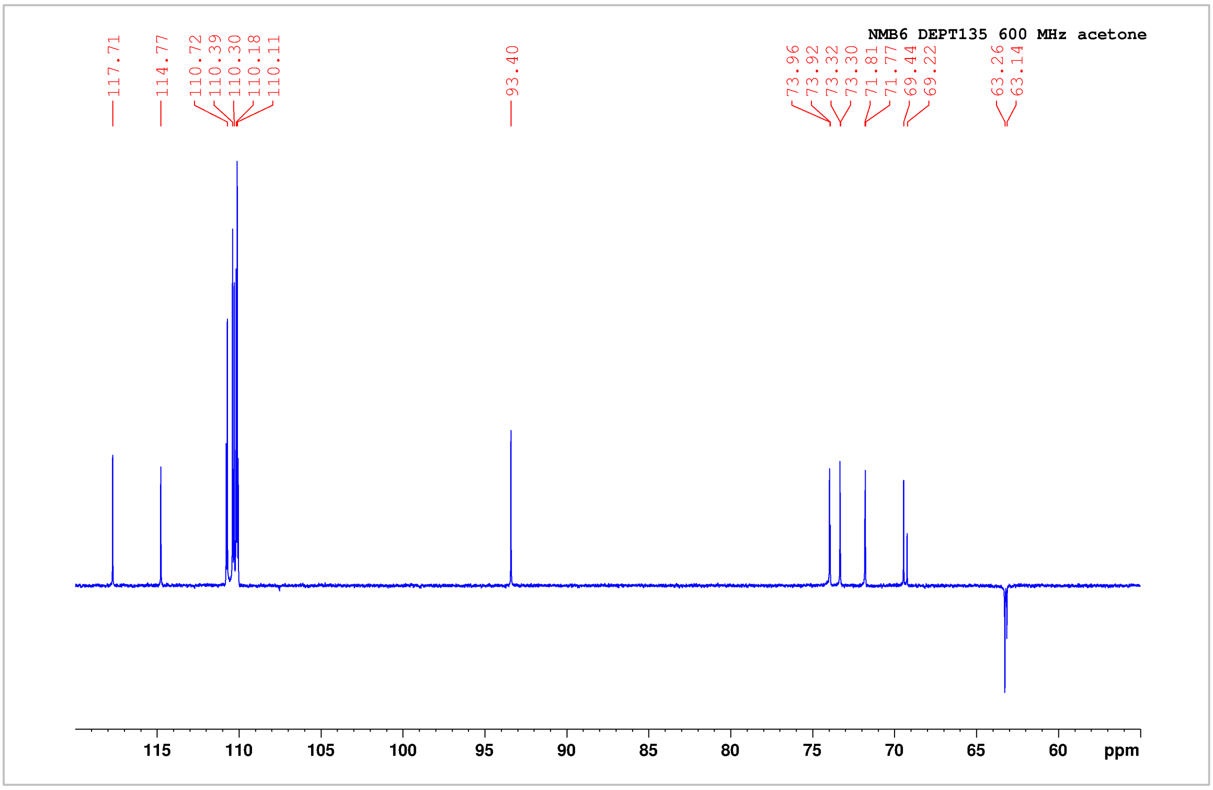

Figure S29: DEPT135 spectrum of 6-O-digalloyl-1,2,3,4-tetra-O-galloyl-β-D-glucose (NMB6)

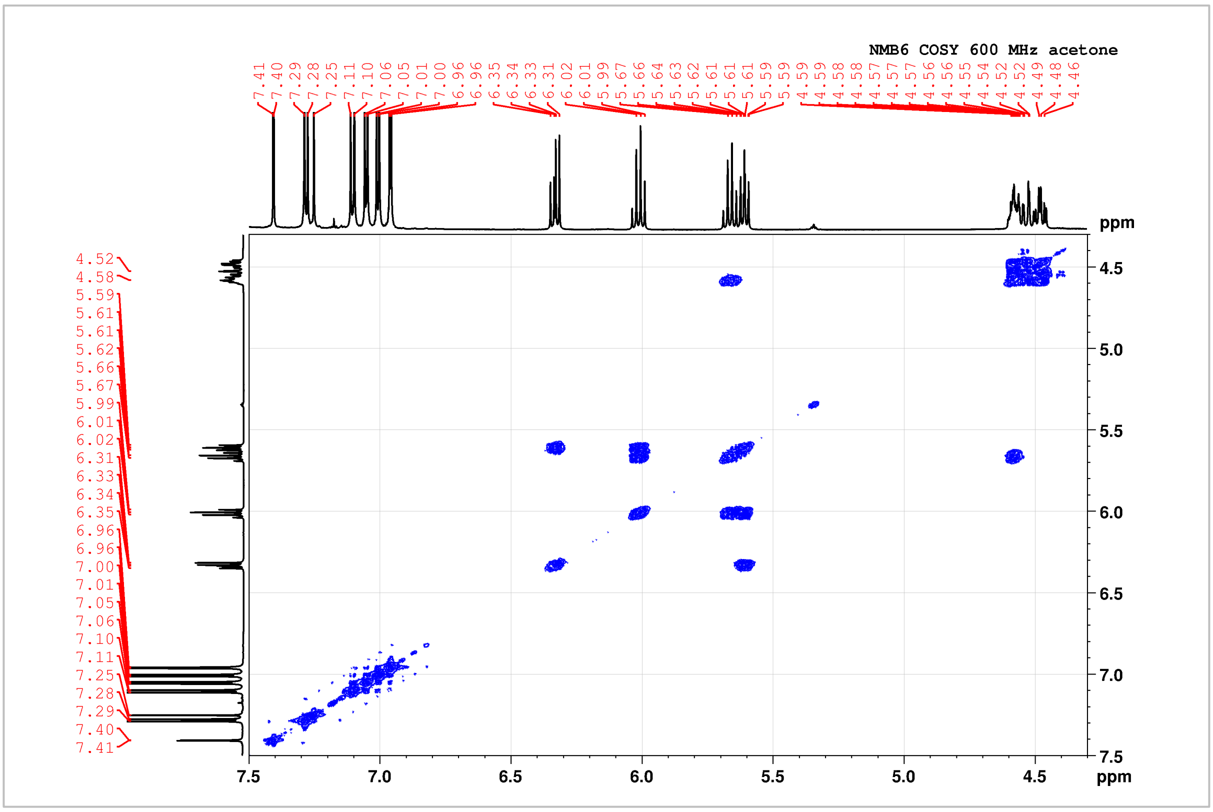

Figure S30: COSY spectrum of 6-O-digalloyl-1,2,3,4-tetra-O-galloyl-β-D-glucose (NMB6)

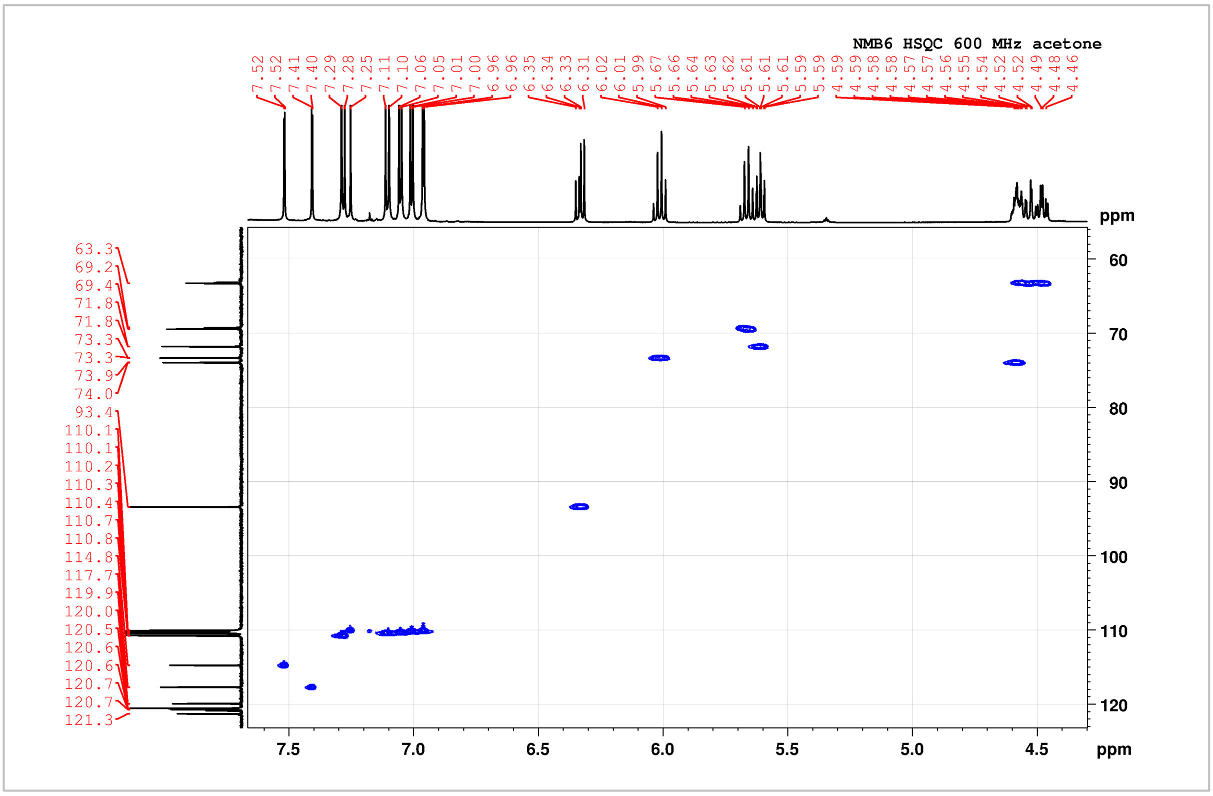

Figure S31: HSQC spectrum of 6-O-digalloyl-1,2,3,4-tetra-O-galloyl-β-D-glucose (NMB6)

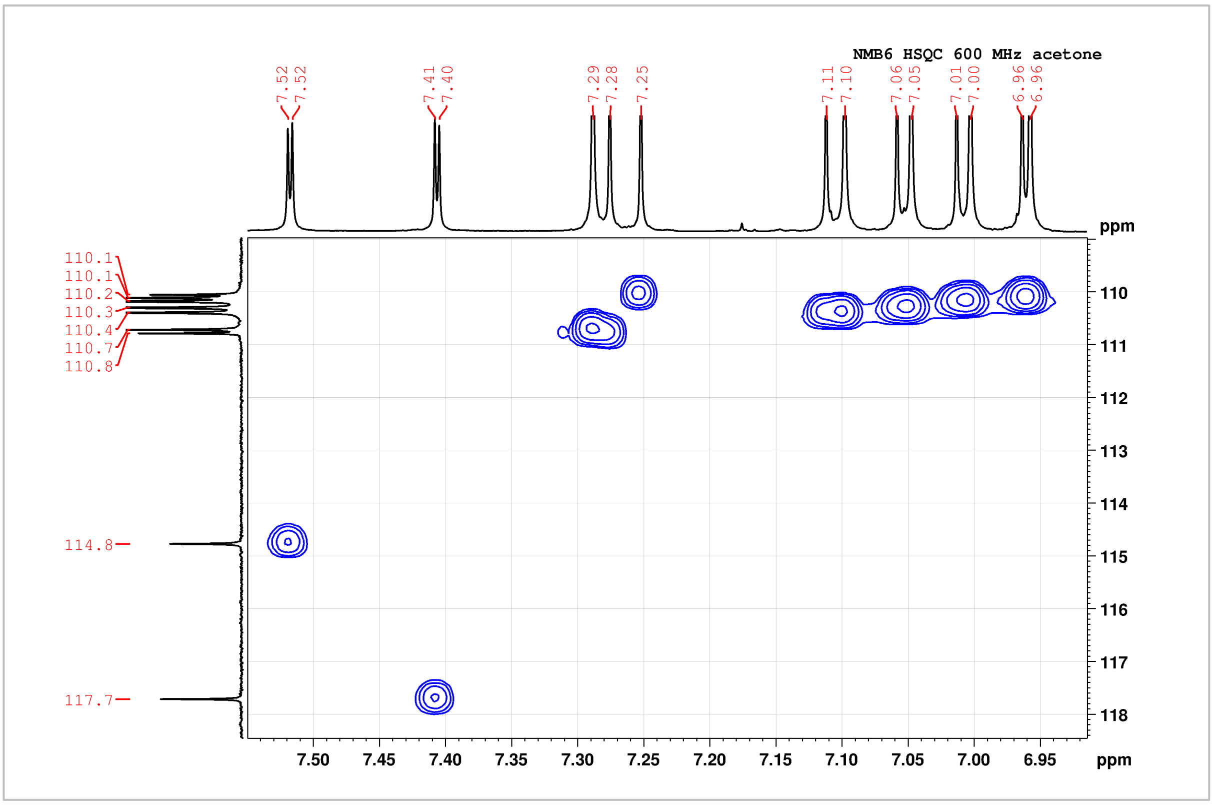

Figure S32: Zoom in of the aromatic region of HSQC spectrum of 6-O-digalloyl-1,2,3,4-tetra-O-galloyl-β-D-glucose (NMB6)

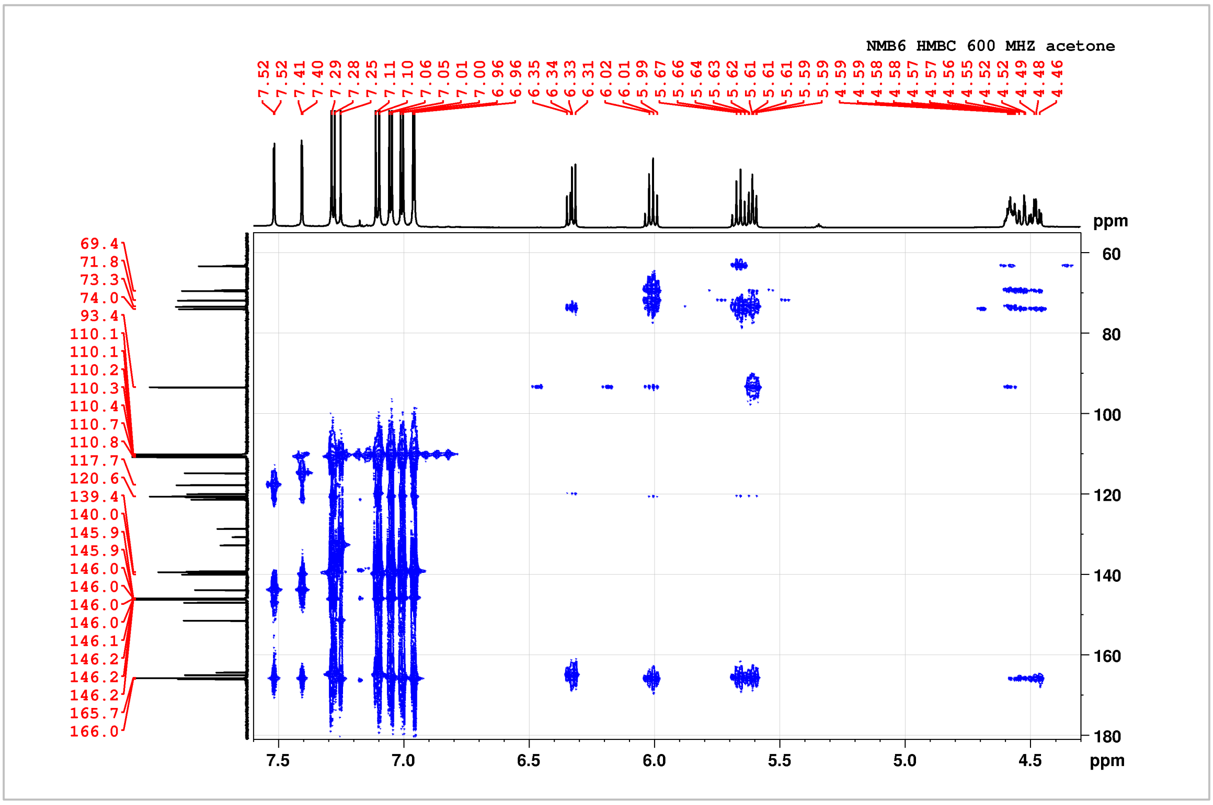

Figure S33: HMBC spectrum of 6-O-digalloyl-1,2,3,4-tetra-O-galloyl-β-D-glucose (NMB6)

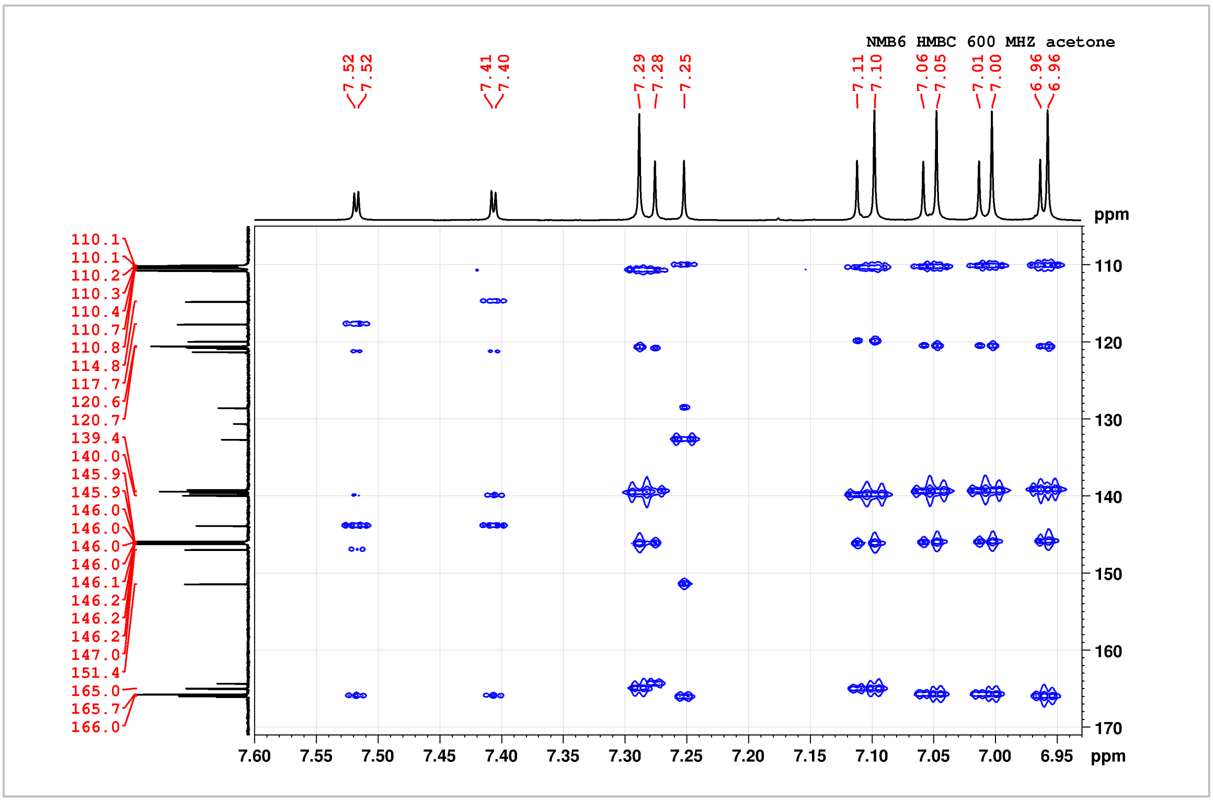

Fig S34: Zoom in of the aromatic region of HMBC spectrum of 6-O-digalloyl-1,2,3,4-tetra-O-galloyl-β-D-glucose (NMB6)

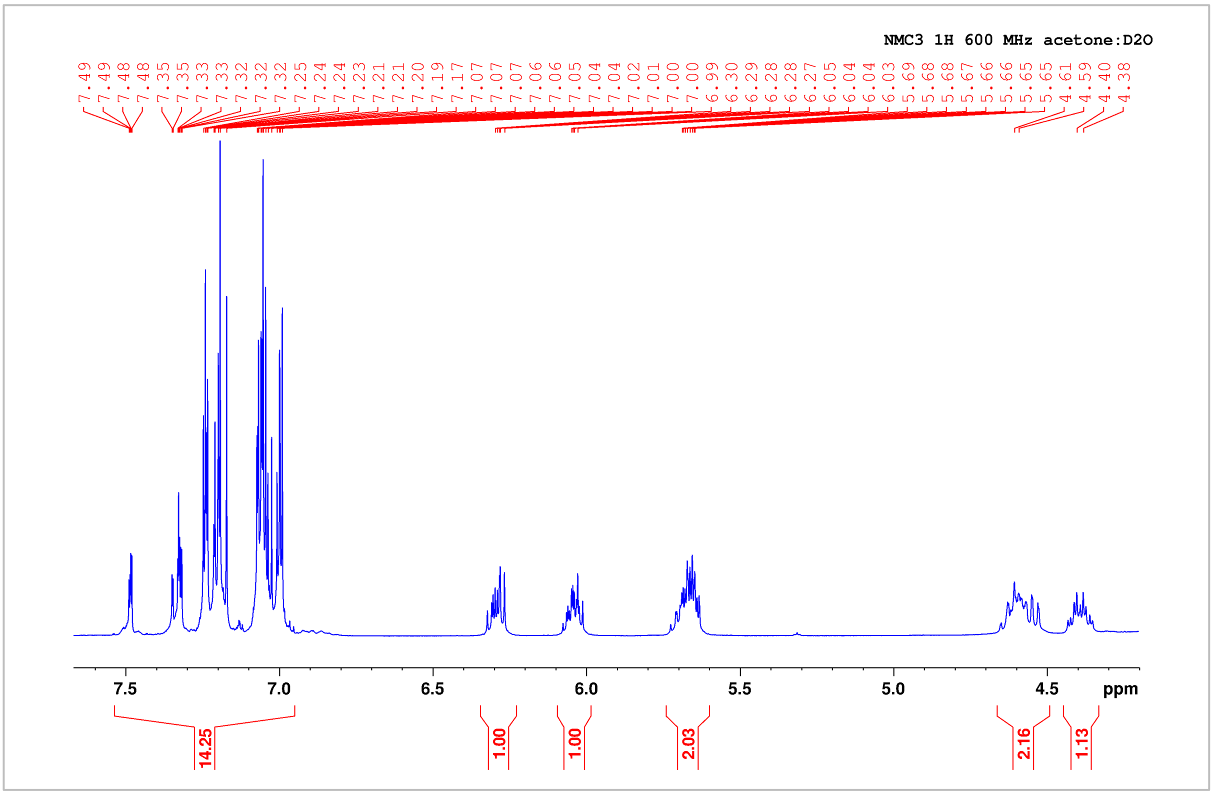

Figure S35: ^{1}H spectrum of 3,6-bis-O-digalloyl-1,2,4-tri-O-galloyl-β-D-glucose (NMC3)

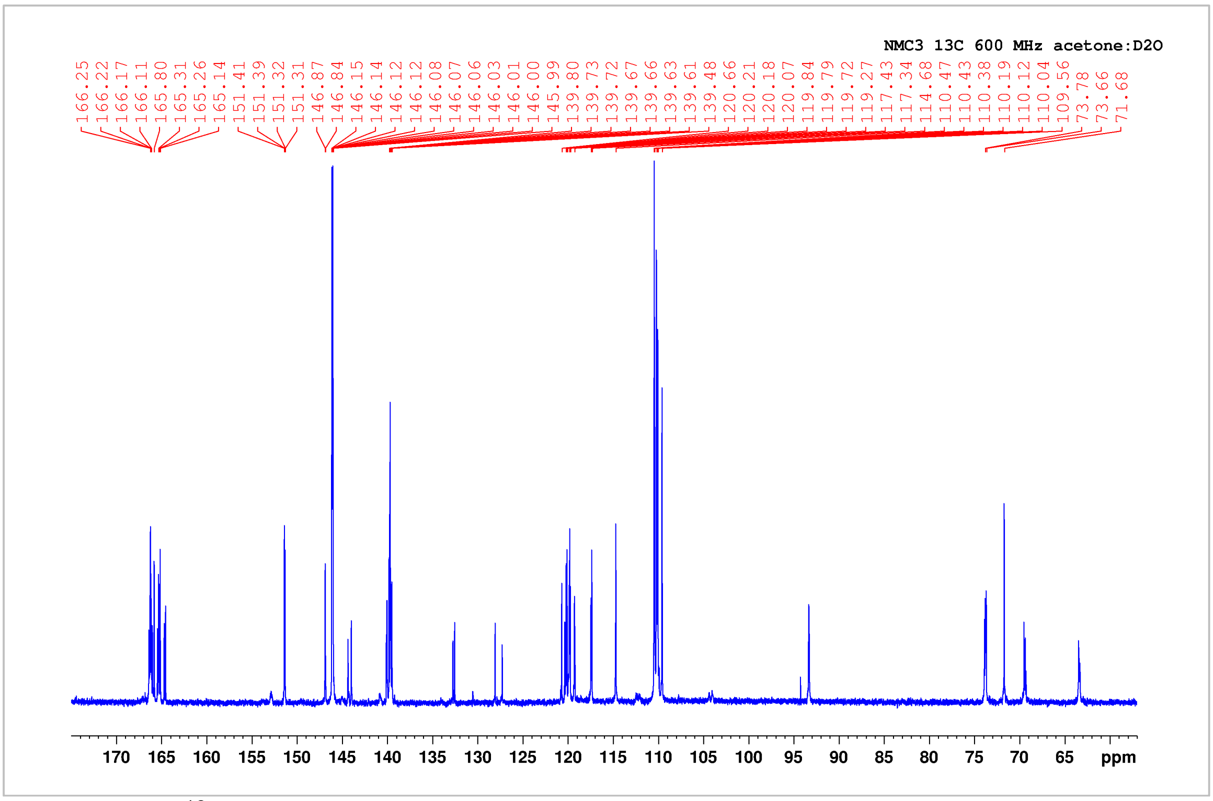

Figure S36: ^{13}C spectrum of 3,6-bis-O-digalloyl-1,2,4-tri-O-galloyl-β-D-glucose (NMC3)

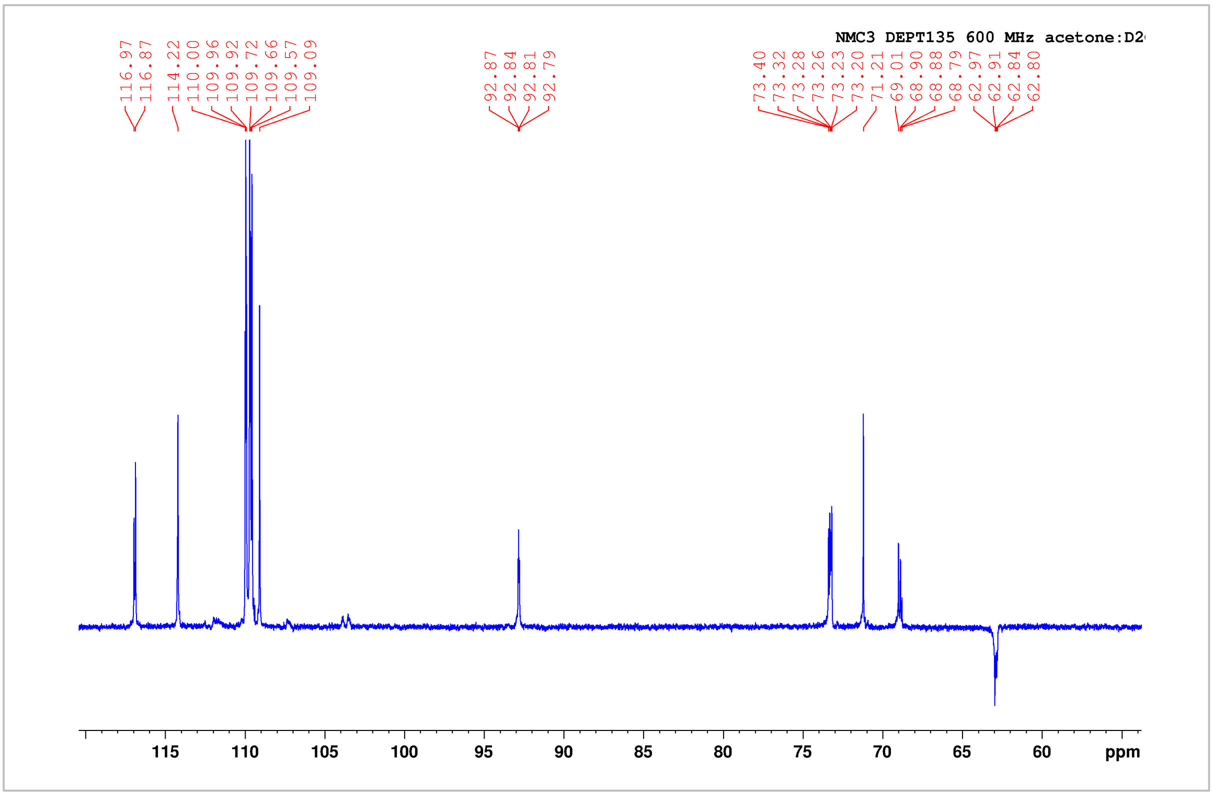

Figure S37: DEPT-135 spectrum of 3,6-bis-O-digalloyl-1,2,4-tri-O-galloyl-β-D-glucose (NMC3)

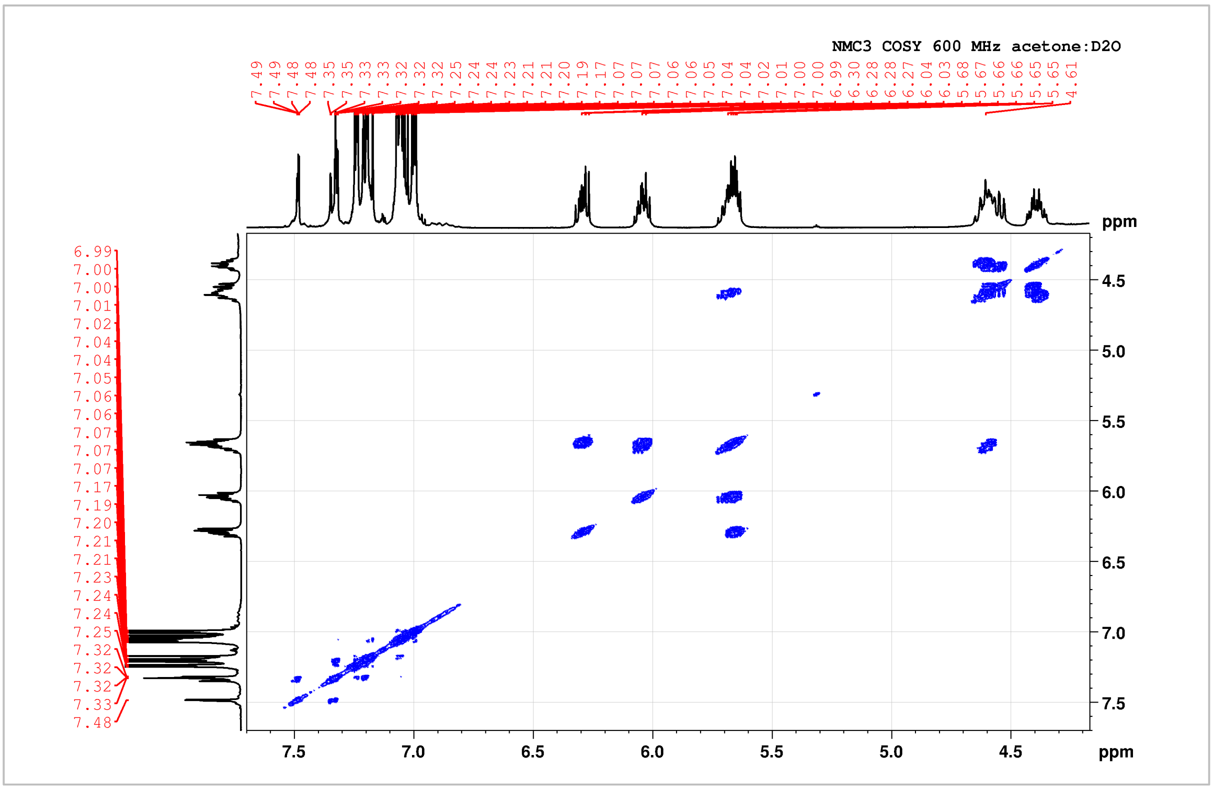

Figure S38: COSY spectrum of 3,6-bis-O-digalloyl-1,2,4-tri-O-galloyl-β-D-glucose (NMC3)

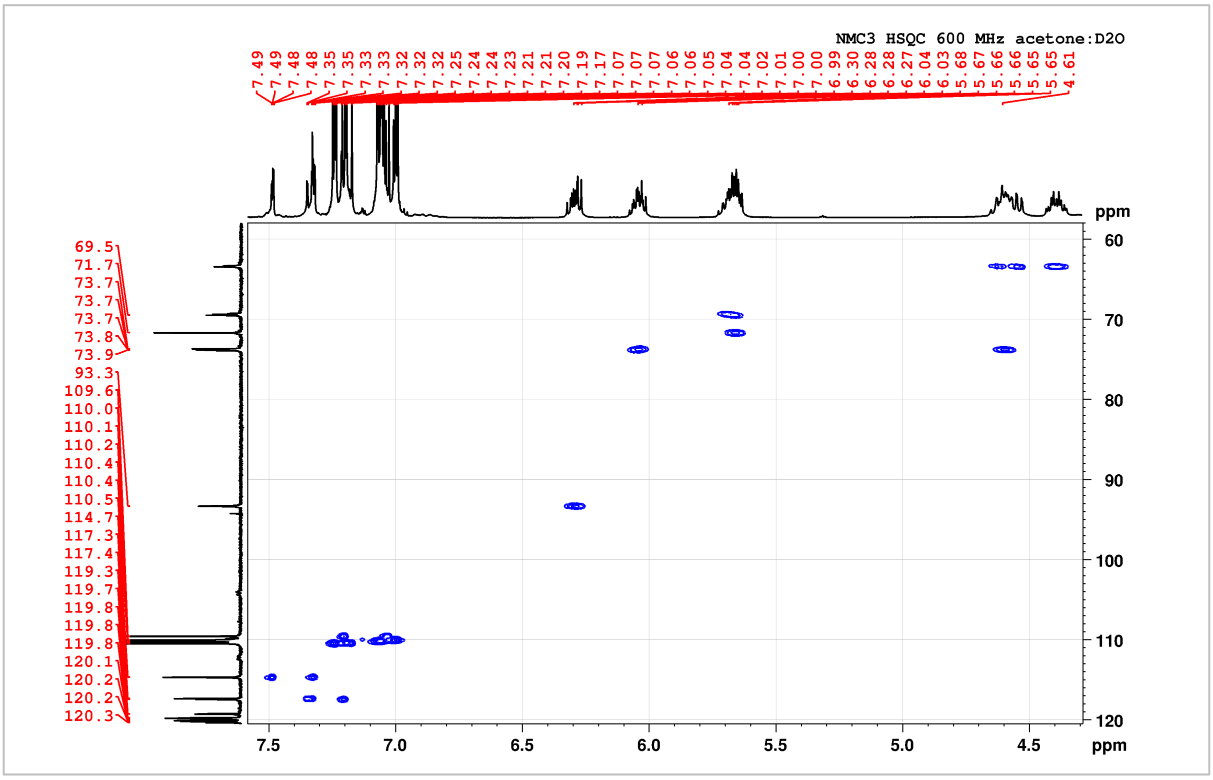

Figure S39: HSQC spectrum of 3,6-bis-O-digalloyl-1,2,4-tri-O-galloyl-β-D-glucose (NMC3)

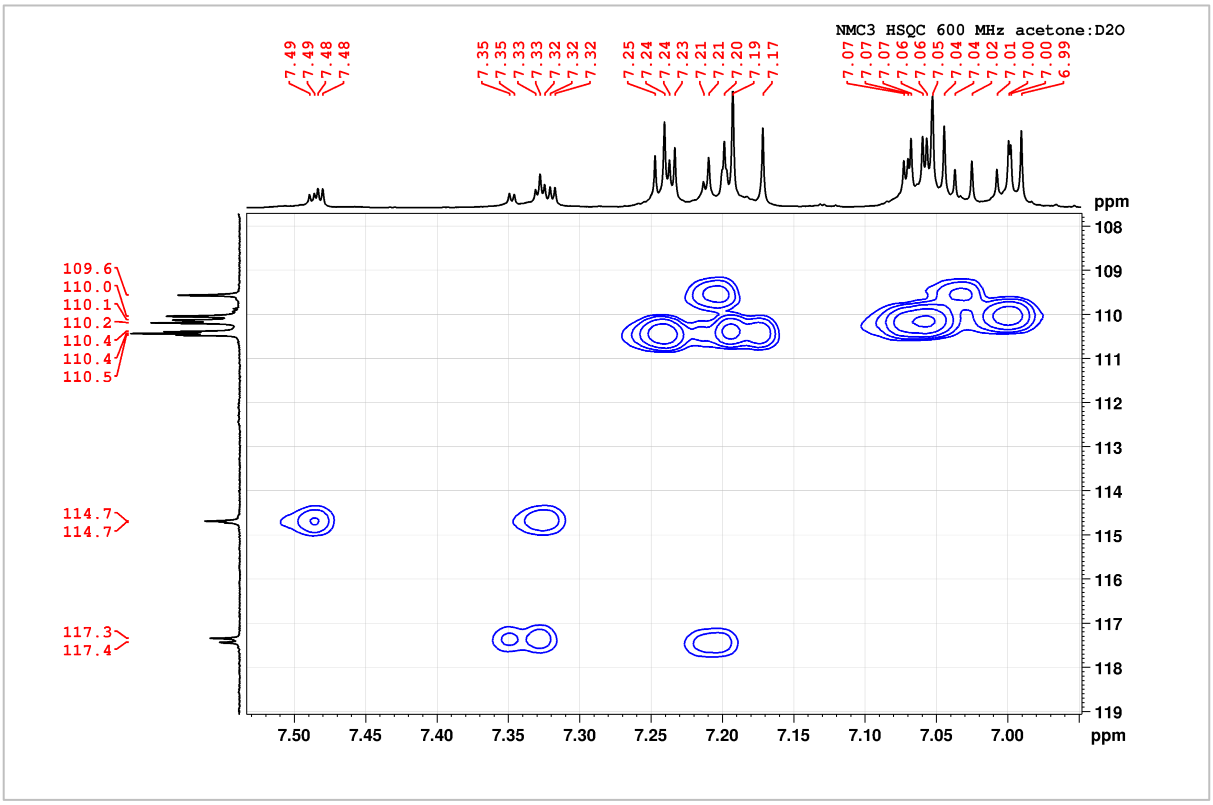

Figure S40: Zoom in of the aromatic region of the HSQC spectrum of 6-O-digalloyl-1,2,3,4-tetra-O-galloyl-β-D-glucose (NMB6)

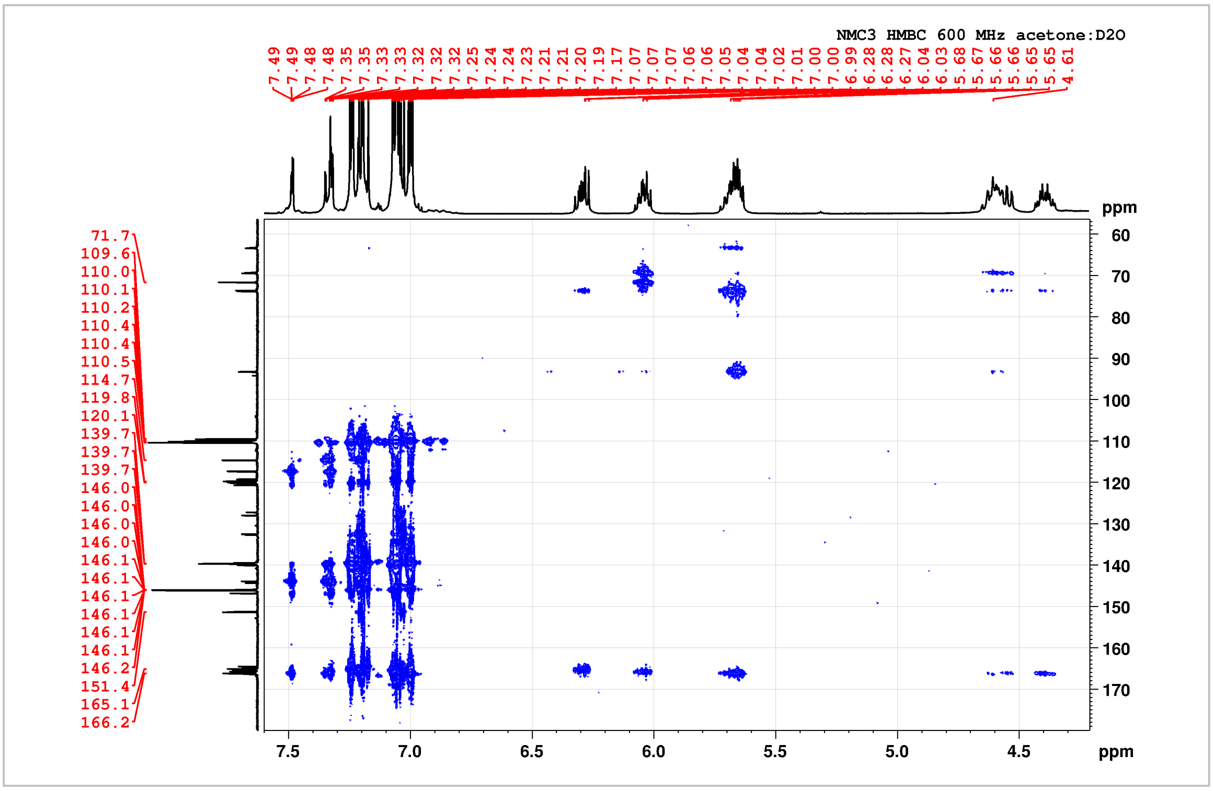

Figure S41: HMBC spectrum of 3,6-bis-O-digalloyl-1,2,4-tri-O-galloyl-β-D-glucose (NMC3)

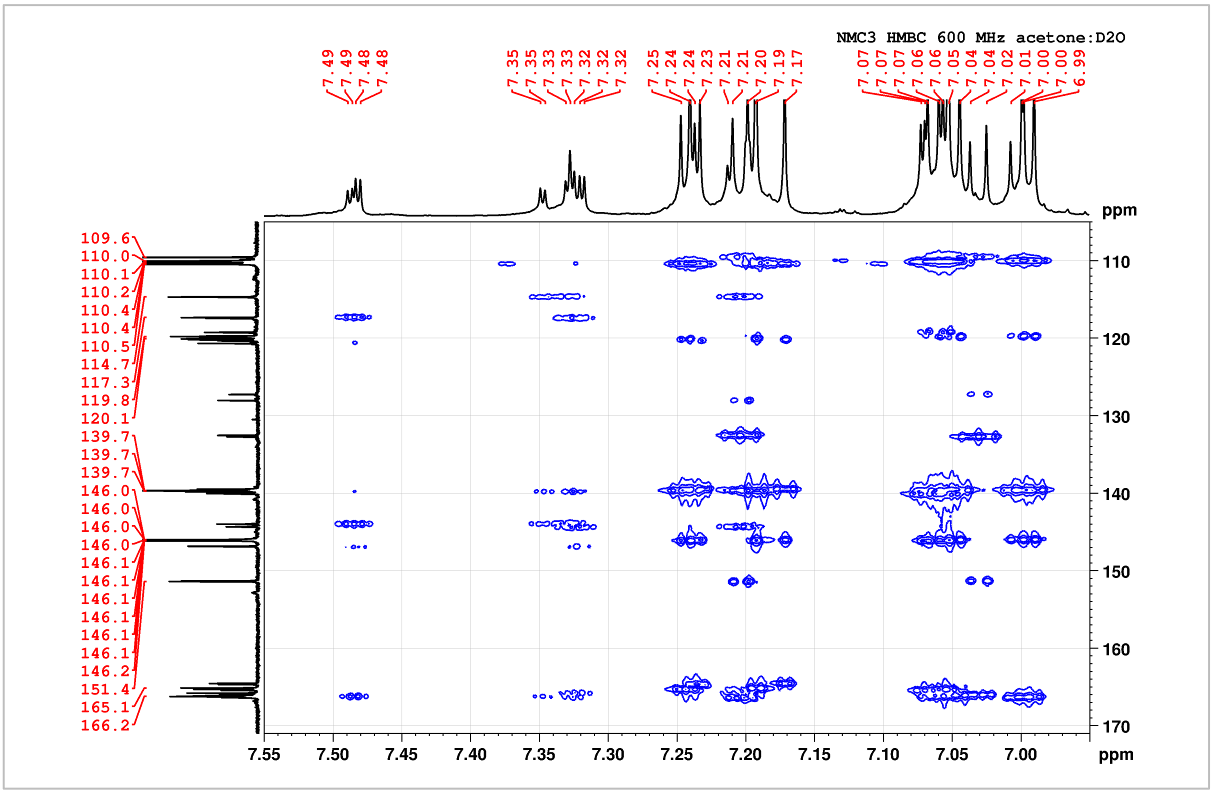

Figure S42: Zoom in of the aromatic region of HMBC spectrum of 3,6-bis-O-digalloyl-1,2,4-tri-O-galloyl-β-D-glucose (NMC3)
